# All-in-One: A Multifunctional Composite Biomimetic Cryogel for Coagulation Disorder Hemostasis and Infected Diabetic Wound Healing

**DOI:** 10.1007/s40820-024-01603-1

**Published:** 2025-03-03

**Authors:** Jiaxin Wang, Yutong Yang, Huiru Xu, Shengfei Huang, Baolin Guo, Juan Hu

**Affiliations:** 1 Department of Otorhinolaryngology, Head and Neck Surgery, The Second Affiliated Hospital of Xi’an Jiaotong University, Xi’an, 710004 People’s Republic of China; 2 State Key Laboratory for Mechanical Behavior of Materials, Key Laboratory of Shaanxi Province for Craniofacial Precision Medicine Research, College of Stomatology, Frontier Institute of Science and Technology, Xi’an Jiaotong University, Xi’an, 710049 People’s Republic of China

**Keywords:** Gelatin-based cryogel, Coagulopathic bleeding, Non-compressible hemorrhage, Diabetic wound healing

## Abstract

Traditional hemostatic materials are difficult to meet the needs of non-compressible bleeding and for coagulopathic patients. In addition, open wounds are susceptible to infection, and then develop into chronic wounds. However, the development of integrated dressings that do not depend on coagulation pathway and improve the microenvironment of chronic wounds remains a challenge. Inspired by the porous structure and composition of the natural extracellular matrix, adipic dihydrazide modified gelatin (GA), dodecylamine-grafted hyaluronic acid (HD), and MnO_2_ nanozyme (manganese dioxide)@DFO (deferoxamine)@PDA (polydopamine) (MDP) nanoparticles were combined to prepare GA/HD/MDP cryogels through amidation reaction and hydrogen bonding. These cryogels exhibited good fatigue resistance, photothermal antibacterial (about 98% killing ratios of both *Escherichia coli* and methicillin-resistant *Staphylococcus aureus* (MRSA) after 3 min near-infrared irradiation), reactive oxygen species scavenging, oxygen release, and angiogenesis properties. Furthermore, in the liver defect model of rats with coagulopathy, the cryogel displayed less bleeding and shorter hemostasis time than commercial gelatin sponge. In MRSA-infected diabetic wounds, the cryogel could decrease wound inflammation and oxidative stress, alleviate the hypoxic environment, promote collagen deposition, and induce vascular regeneration, showing a better repair effect compared with the Tegaderm™ film. These results indicated that GA/HD/MDP cryogels have great potential in non-compressible hemorrhage for coagulopathic patients and in healing infected wounds for diabetic patients.

**Supplementary Information:**

The online version contains supplementary material available at 10.1007/s40820-024-01603-1.

## Highlights

- An all-in-one cryogel is proposed for hemostasis of coagulopathy bleeding and repair of infected diabetic ulcers.
- The cryogel possesses rapid liquid-triggered volume expansion capability and physicochemical synergistic hemostatic properties.
- The cryogel can accelerate the healing of infected diabetic wounds by releasing oxygen and deferoxamine.

**Supplementary Information:**

The online version contains supplementary material available at 10.1007/s40820-024-01603-1.

## Graphical abstract

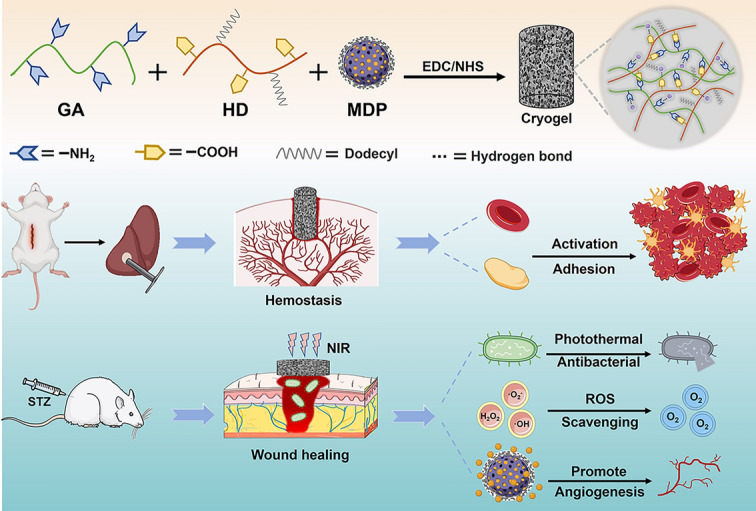

## Introduction

Wound hemostasis and wound healing are always the major public health issues with global concern. More than 5.8 million people die globally each year caused by severe trauma such as wars and accidents [1]. The main cause of death is because of bleeding from non-compressible blooding [2]. In addition, one-quarter of severely traumatized patients have coagulation disorders in the early stages, resulting in excessive blood loss owing to platelet insufficiency as well as coagulation factor deficiencies, which threaten the patient’s life [3]. Furthermore, open wounds are susceptible to developing into chronic wounds because of local and systemic factors. The diabetic wound is a common and serious chronic wound with bacterial infection, excessive inflammation, oxidative imbalance, hypoxia, and vascular network destruction [4]. These factors lead to prolonged healing times, imposing a significant economic burden and decreasing the quality of patients’ lives [5]. In summary, the development of multifunctional materials is crucial to address the challenges of hemostasis in non-compressible bleeding among coagulation-disordered patients and to promote healing in chronic wounds among diabetic patients.

Different forms of materials have been developed to address the above challenges. Cryogel, with its interconnected porous structure, can effectively concentrate blood during hemostasis and provide a moist and breathable environment during healing, which makes it an ideal dressing form [6, 7]. However, for coagulation-disordered patients, simple physical sealing is not effective in hemostasis, and it is difficult to rely on biological and chemical strategies to activate the coagulation pathway resulting in synergistic hemostasis [8]. Therefore, researchers have proposed that aggregating and activating red blood cells and platelets can promote blood clot formation independent of coagulation cascades reaction, which in turn accelerates coagulation [9]. Among the various components that interact with blood cells, the dodecyl hydrophobic chain can insert and anchor to the lipid bilayer of cell membrane, inducing the aggregation of red blood cells and platelets through hydrophobic interactions [10, 11]. Furthermore, the catechol group of polydopamine (PDA) can interact covalently and non-covalently with the reactive groups of proteins or polysaccharides in blood cells [8]. It effectively adheres and activates red blood cells and platelets, which in turn accelerates clot formation [12, 13]. We hypothesize that introducing hydrophobic chains and catechol groups into cryogels can make them independent of coagulation factors, and effectively enhance the hemostatic effect of non-compressible bleeding in coagulopathic patients through physical and chemical effects.

Diabetic wounds with persistently high blood glucose levels present a complex microenvironment that demands antibacterial, reactive oxygen species (ROS) scavenging, and vascular reconstruction to promote healing [14]. Studies have shown that natural antioxidant enzymes generated by the organism are effective in maintaining an oxidative balance in the body [15]. However, these natural enzymes are susceptible to inactivation in the harsh wound microenvironment [16], limiting their clinical application. In recent years, the development of nanozymes has overcome the disadvantages of natural enzymes [17]. Among various nanozymes, MnO_2_ exhibits superoxide dismutase (SOD) and catalase (CAT)-like activity, which converts ·O_2_^−^ to H_2_O_2_ and O_2_, and decomposes H_2_O_2_ to H_2_O and O_2_, respectively, improving wound hypoxia issues while decreasing the level of oxidative stress [18, 19]. Furthermore, MnO_2_ has broad absorption in the near-infrared (NIR) range [20, 21], which could endow the dressing with the photothermal antibacterial properties. For vascular regeneration, the small molecule drug deferoxamine (DFO), an FDA-approved hypoxia simulant, could inhibit prolyl hydroxylase and stable hypoxia-inducible factor-1, as well as up-regulate the expression of VEGF and other key angiogenesis-related factors to promote angiogenesis [22, 23]. However, using MnO_2_ to construct materials with integrated photothermal stability, ROS scavenging persistence, and drug-sustained release is a challenge.

Herein, based on the functional properties of gelatin and hyaluronic acid in the composition of the extracellular matrix, we prepared a multifunctional cryogel based on adipic dihydrazide-grafted gelatin (GA), dodecylamine-grafted hyaluronic acid (HD) as polymer composition, in the hope of achieving optimal functionalization by maximally mimicking the composition and function of the extracellular matrix. Meanwhile, the PDA-coated and DFO-loaded MnO_2_ nanoparticles (MDP) as a functional composite nanozyme achieve the integration of multiple antioxidant activities and pro-angiogenesis. The cryogel integrated hemostasis, photothermal antibacterial, ROS scavenging, oxygen release, and angiogenesis properties to promote hemostasis in coagulation-disordered patients and wound repair in diabetic patients. We evaluated the biocompatibility, in vitro blood clotting, photothermal antibacterial, antioxidant, oxygen and DFO release properties of GA/HD/MDP cryogels. Additionally, the hemostatic ability of cryogel was compared with the commercial gelatin sponge by the Sprague Dawley (SD) rat liver hemorrhage model with coagulation disorder. Finally, a full-thickness skin defect model was further established in methicillin-resistant *Staphylococcus aureus* (MRSA)-infected diabetic mice to demonstrate the application potential of GA/HD/MDP cryogel by evaluating the wound closure ratio, inflammatory infiltration, collagen deposition, and angiogenesis levels. The study results revealed the potential of the GA/HD/MDP cryogels for applications in coagulation-disordered patients’ rapid hemostasis and diabetic patients infected wound repair.

## Experiment Section

### Synthesis of GA, HD, and MDP Nanoparticles (NPs)

GA, HD, and MDP nanoparticles were synthesized according to the previous work [18, 24, 25]. Briefly, gelatin (Gel) and hyaluronic acid (HA) were modified using adipic dihydrazide (ADH) and dodecylamine (DDA), respectively. Subsequently, SiO_2_ was used as a template agent to reduce KMnO_4_ and form hollow MnO_2_ microspheres in situ. Then, DFO was loaded inside, and PDA was coated outside to prepare MDP nanoparticles. Details of the synthesis steps are described in Supporting Information.

### Synthesis of GA/HD/MDP Cryogel

**Fig. 1:**
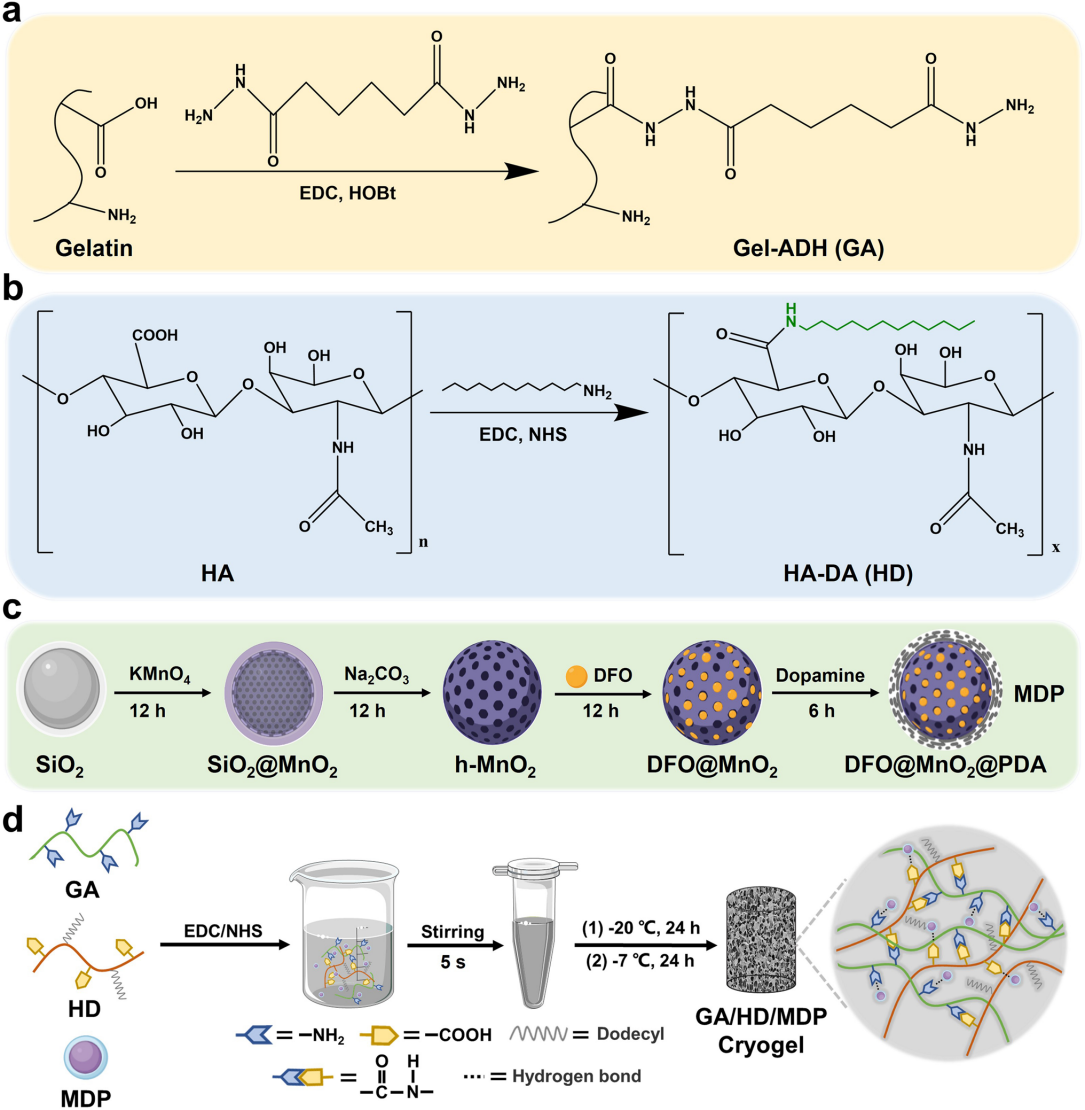
Synthesis of **a** GA, **b** HD and **c** MDP nanoparticles. **d** Preparation scheme of the GA/HD/MDP cryogel

**Fig. 2:**
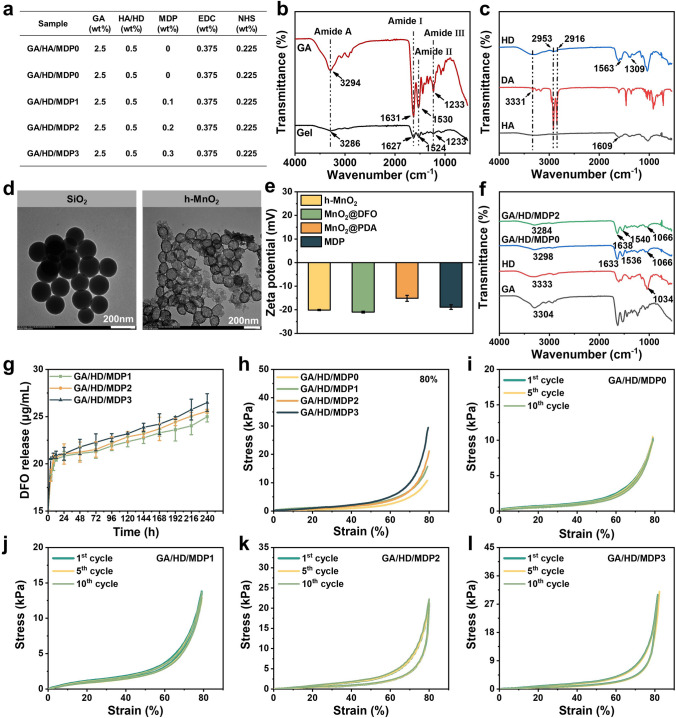
**a** Concentration of GA/HD/MDP cryogels components. FT-IR spectra of **b** GA and **c** HD. **d** TEM images of SiO_2_ and h-MnO_2_. **e** Zeta potential of h-MnO_2_, MnO_2_@DFO, MnO_2_@PDA, and MnO_2_@DFO@PDA (MDP). **f** FT-IR spectra of GA/HD/MDP cryogels. **g** DFO release of GA/HD/MDP cryogels. **h** Uniaxial compression stress–strain curves of the cryogels at 80% strain. The cyclic compression stress–strain curves at 80% strain of **i** GA/HD/MDP0, **j** GA/HD/MDP1, **k** GA/HD/MDP2, and **l** GA/HD/MDP3 cryogels

Firstly, GA concentration was fixed at 6.25 wt% according to the publication [26, 27], and HD was dissolved in deionized water to form a 1.25 wt% homogeneous solution. Then, MDP nanoparticles were added to deionized water to form 10, 20, and 30 mg mL^−1^ dispersions, which were uniformly dispersed under ultrasound. Subsequently, GA, HD, MDP, EDC (75 mg mL^−1^), and NHS (45 mg mL^−1^) solutions were pre-chilled in an ice-water mixture for 2 h. Later, the 400 μL GA and 400 μL HD were mixed with 100 μL MDP dispersions, and then the EDC (50 μL) and NHS (50 μL) were added. The combination was immediately immersed in iced ethanol (-20 °C) for 24 h and later transferred to a -7 °C refrigerator for 24 h to obtain GA/HD/MDP cryogels (Fig. 1d). The parameter of GA/HD/MDP cryogels is shown in Fig. 2a.

### Characterizations

^1^H nuclear magnetic resonance (^1^H NMR) (Bruker Ascend 400 MHz) was used to confirm the structure of GA. The structures of the GA, HD, and GA/HD/MDP cryogels were confirmed using Fourier transform infrared spectroscopy (FT-IR) (Bruker ALPHA II). Transmission electron microscope (TEM), particle size and zeta potential analyzer (Zetasizer Pro) were used to characterize the MDP nanoparticles. Scanning electron microscope (SEM; QUTAN FEG 250, FEI) was used to observe the morphology of the GA/HD/MDP cryogels. A microplate reader (Molecular Devices) was used to research the loading and release of DFO. Rotational rheometer (TA) was used to research the mechanical properties of the cryogels. Additionally, the cryogels’ abilities for absorbing blood and water were evaluated. Detailed steps are shown in Supporting Information.

### Photothermal Property of the Cryogels

Firstly, the freeze-dried cryogels were prepared into cylindrical shapes of the same size. After reaching equilibrium swelling in deionized water, the cryogels were irradiated for 10 min under a NIR laser. The temperature at each time point was recorded by an IR thermal camera. Detailed steps are shown in Supporting Information.

### Antibacterial Property of the Cryogels

The cryogel samples were prepared into disks with a diameter of 8 mm and a height of 5 mm, and the photothermal antibacterial activity of the cryogels was evaluated by using *Escherichia coli* (*E. coli*) and methicillin-resistant *Staphylococcus aureus* (MRSA). Detailed steps are shown in Supporting Information.

### Antioxidant of the Cryogels

The antioxidant properties of the GA/HD/MDP cryogels were evaluated by H_2_O_2_ scavenging, ·OH scavenging, ·O_2_^−^ scavenging, and cellular antioxidant assays. Detailed steps are shown in Supporting Information.

### Oxygen Release Properties of Cryogel

Firstly, GA/HD/MDP cryogels were placed in deionized water containing H_2_O_2_, and then the concentration of O_2_ at the pre-set time was recorded using the portable dissolved oxygen analyzer (JPBJ-608, Leimagnet, Shanghai). Detailed steps are shown in Supporting Information.

### Biocompatibility of the Cryogels

Good biocompatibility is essential for biomaterials. The biosafety of GA/HD/MDP cryogels was evaluated by hemocompatibility, cytocompatibility and *in vivo* biocompatibility. Detailed steps are shown in Supporting Information.

### In Vitro Hemostatic Performance Evaluation

Firstly, the cryogel samples were prepared into disks with the identical size. The *in vitr*o clotting ability of the GA/HD/MDP cryogels was evaluated qualitatively and quantitatively by the red blood cell and platelet adhesion assays as well as the dynamic whole blood clotting assay, respectively. Detailed steps are shown in Supporting Information.

### In Vivo Hemostatic Performance Evaluation

Firstly, cylindrical cryogel samples with the same size were sufficiently compressed and fixed. Subsequently, the in vivo hemostatic properties of the GA/HD/MDP cryogels were evaluated by establishing liver defect models in normal SD rats and coagulopathic SD rats. Detailed steps are shown in Supporting Information.

### In Vivo Wound Healing Performance Evaluation

The wound repair effect of GA/HD/MDP cryogel was evaluated by establishing the model of MRSA-infected diabetic mice with full-thickness skin defects. On day 5, 10, and 21, the wound sites were photographed and sampled. Epidermal regeneration, inflammatory infiltration, and collagen deposition at the wound site were estimated by H&E and Masson staining. Meanwhile, CD80 and CD206 immunofluorescence staining was used to observe the inflammatory response of the wounds in each group, and the angiogenesis of the wounds in each group was investigated by VEGF immunofluorescence staining. Detailed steps are shown in Supporting Information.

### Statistical Analysis

The experimental data were analyzed using one-way ANOVA and presented as mean ± standard deviation. Statistically significant differences were indicated by the symbols *P (< 0.05), **P (< 0.01), and ***P (< 0.001).

## Results and Discussions

### Preparation and Characterization of the GA, HD, and MDP NPs

Before the preparation of cryogels, gelatin and hyaluronic acid were modified by ADH and dodecylamine (DDA), respectively (Fig. 1a, b). The structure of GA was characterized by ^1^H NMR and FT-IR. Figure S1 shows that the peaks at 2.18 and 1.58 ppm were attributed to the methylene peak of ADH [25]. Meanwhile, in the FT-IR spectrum (Fig. 2b), the increase in specific amide signal peaks at 1233 cm^−1^ (amide III), 1530 cm^−1^ (amide II), and 1631 cm^−1^ (amide I) also synergistically indicated the successful modification of ADH [28]. As shown in Fig. 2c, the absorption peaks at 2953 and 2916 cm^−1^ corresponded to the stretching vibration of C-H on the alkyl chain, while the absorption peaks of N–H stretching vibration at 3331 cm^−1^ and C–N–H bending vibration at 1563 cm^−1^ were enhanced, indicating the successful grafting of the dodecyl chain. In addition, the shift of C = O stretching vibration peak on HD and the enhancement of C–N stretching characteristic peak at 1309 cm^−1^ (amide III) synergistically confirmed the successful synthesis of HD [29].

Subsequently, MDP nanoparticles with photothermal, antioxidant, and oxygen release properties were synthesized (Fig. 1c). Firstly, hollow MnO_2_ (h-MnO_2_) microspheres were formed by in situ reduction of KMnO_4_ with SiO_2_ as a template. The morphology and particle size of h-MnO_2_ were observed by TEM. As shown in Fig. 2d, SiO_2_ exhibited a smooth surface, whereas the deposition of MnO_2_ resulted in a rough surface and formed a distinct hollow structure. The particle size of h-MnO_2_ ranges from 110 to 190 nm, with an average particle size slightly larger than that of SiO_2_ (Fig. S2a, b). The above results confirmed the successful synthesis of h-MnO_2_. Secondly, the pro-angiogenic drug DFO was loaded into the h-MnO_2_. The concentration of DFO before and after encapsulation was calculated, and the results showed that the encapsulation efficiency of DFO was about 36.2% (Fig. S3). Finally, the outer surface of the MnO_2_@DFO was coated with polydopamine, which could activate and adhere to the platelets and red blood cells. The particle size and zeta potential of h-MnO_2_, MnO_2_@DFO, MnO_2_@PDA, and MnO_2_@DFO@PDA (MDP) were tested. As shown in Fig. S4a, b, the average particle sizes of h-MnO_2_ and MnO_2_@DFO were 164 and 190 nm, respectively. After PDA coating, the particle sizes of MnO_2_@PDA and MDP increased to 341 and 396 nm, respectively (Fig. S4c, d). Zeta potential results (Fig. 2e) indicated that the surface of h-MnO_2_ nanoparticles possessed a negative charge even after loading DFO, and there was no significant change between h-MnO_2_ and MnO_2_@DFO. However, after in situ polymerization of DA on the surface of MnO_2_@DFO, the zeta potential increased from −21 to −18 mV. This indicated that PDA was successfully modified onto the surface of MnO_2_@DFO through electrostatic interactions. The above results synergistically proved the successful preparation of MDP nanoparticles.

### Preparation and Characterization of the GA/HD/MDP Cryogel

The three-dimensional network of cryogels was fabricated through an amidation reaction between GA and HD. As shown in Fig. 2f, compared with the FT-IR spectrum of GA and HD, the intensity of the absorption peaks at 3298 cm^−1^ (N–H stretching vibration) and 1066 cm^−1^ (C–O stretching vibration) in the GA/HD/MDP0 were weakened, which may be due to the amidation reaction of -NH_2_ in GA and –COOH in HD [30]. The ratio of amino and carboxyl groups played a crucial role in determining the cross-linking degree, which directly influenced the performance of the cryogels. Therefore, based on fixed GA concentration (2.5 wt%), the properties of cryogels with different HD concentrations (0.25, 0.5, 1.0, and 1.5 wt%) were explored (Table S1). The swelling test results showed that the cryogel had the highest swelling ratio and short water-absorption time when the HD concentration was 0.5 wt% (Fig. S5). Therefore, the concentration of HD was fixed at 0.5 wt% in the subsequent experiments.

Subsequently, MDP nanoparticles were introduced into the system to endow the cryogel with biological activities such as hemostasis, photothermal antibacterial, antioxidant, and angiogenesis. Comparing the FT-IR spectrum of GA/HD/MDP0 and GA/HD/MDP2 (Fig. 2f), in which the amino characteristic peak shifted from 3298 to 3284 cm^−1^, the carboxy characteristic peak shifted from 1633 and 1536 cm^−1^ to 1638 and 1540 cm^−1^, respectively, which may be caused by the hydrogen bonding between PDA and the amino group on GA as well as the carboxy group on HD interaction [31]. The above results synergistically demonstrated the successful preparation of GA/HD/MDP cryogels. In the following study, the properties of cryogels with different MDP contents (0.1, 0.2, and 0.3 wt%) were further explored.

### DFO Release of the Cryogels

DFO can stabilize and activate hypoxia-inducible factor-1α (HIF-1α), which is associated with the expression of genes related to angiogenesis. The hollow MnO_2_ particles can be used as a carrier for drug loading. Therefore, the DFO release properties of GA/HD/MDP cryogels in PBS (pH 7.4) were tested. As shown in Fig. 2g, the cryogels with different MDP concentrations showed sustained DFO accumulative release behavior within 10 days, and the amount of MDP loaded was positively correlated with the amount of DFO released; thus, the cryogels exhibit a sustained release effect on DFO, making them promise for promoting vascular regeneration during diabetic wound healing.

### Mechanical Performance of the Cryogels

The macroporous structure of cryogels usually accompanies weak mechanical properties. Therefore, MDP nanoparticles were introduced through hydrogen bonding to enhance the mechanical strength and stability of cryogels. Uniaxial compression tests were utilized to verify this point. The results (Fig. 2h) showed that at 80% compressive strain, the compressive stress gradually increased with the MDP concentration. To further evaluate the stability of the GA/HD/MDP cryogels in applications, 10 cycles of compression on different cryogel samples were performed under 80% compressive strain. As shown in Fig. 2i–l, all the cryogels could maintain the original mechanical strength with good stability, which was attributed to the combined chemical and physical cross-linking. Cryogels with good mechanical strength and stability could rapidly concentrate blood and recover their shape to seal the wound in hemostasis and protect the wound in subsequent repair.

### Swelling Ratio, Morphology and Fixed Compression of the Cryogels

**Fig. 3:**
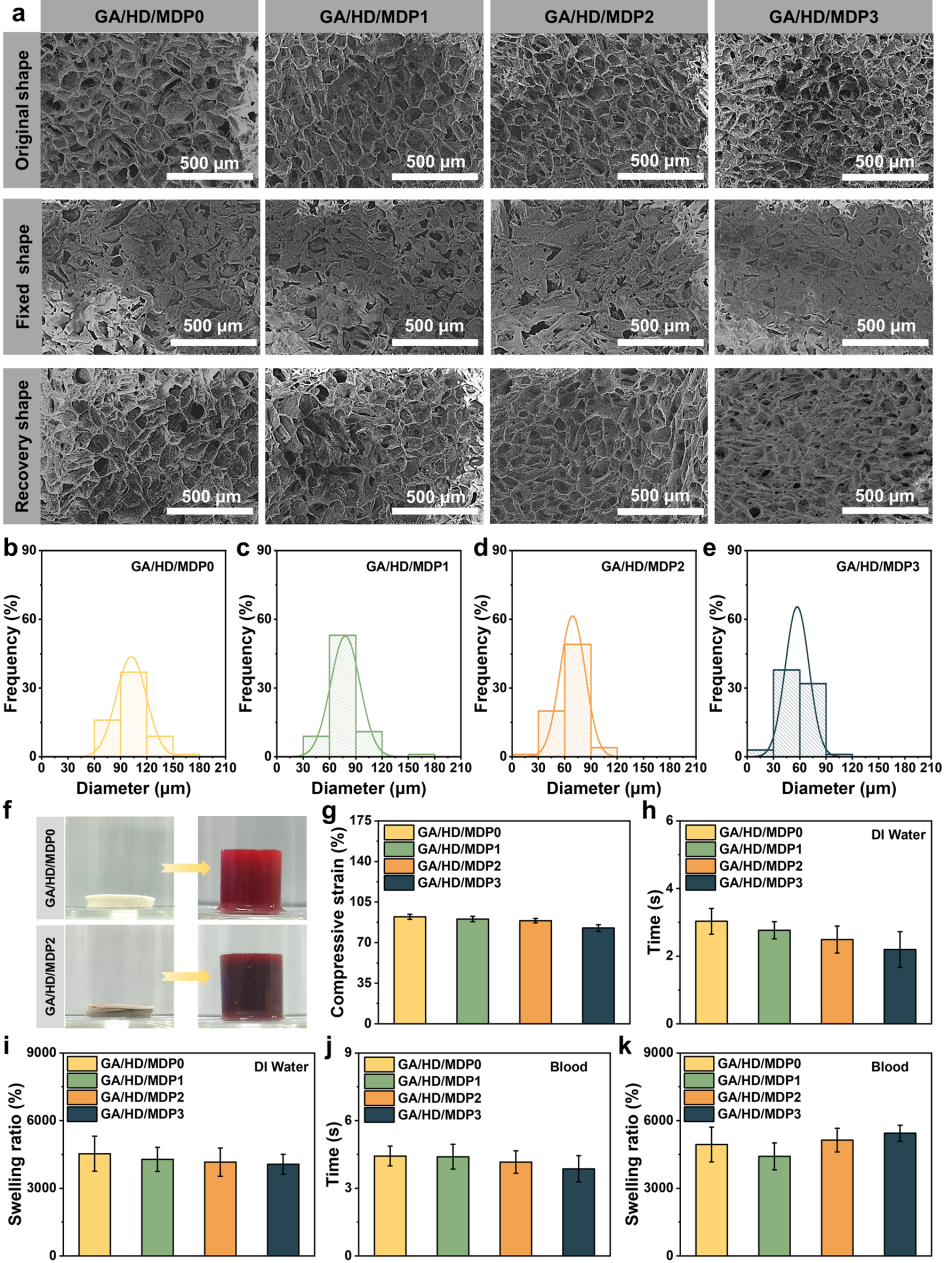
**a** Original pore structure, fixed pore structure, and shape recovery pore structure of GA/HD/MDP cryogels after freeze-drying. Scale bar: 500 μm. The pore diameter distribution of **b** GA/HD/MDP0, **c** GA/HD/MDP1, **d** GA/HD/MDP2, and **e** GA/HD/MDP3 cryogels, respectively. **f** Demonstration of the cryogel swelling before and after blood absorption. **g** Maximum compression of the cryogel. The absorption time and swelling ratio of the cryogels (freeze-dried) in **h, i** deionized water and **j, k** blood, respectively

Water in the pre-polymerization solution formed ice crystals at low temperatures, and the GA and HD slowly cross-linked in the “micro-phase region” to form a three-dimensional network. After the ice crystals melted at room temperature, the cryogel remained with the macroporous structure which allowed the liquid to flow freely, endowing the cryogel with liquid-triggered shape recovery capability. The microstructures of GA/HD/MDP cryogels in the original state, compressed-fixed state, and shape-recovered state were observed by SEM (Fig. 3a). In the original state, all the cryogel groups showed interconnected macroporous structures. The pore diameter of the cryogels was affected by the concentration of MDP nanoparticles, which tended to decrease gradually as the MDP concentration increased from 0 to 0.1, 0.2, and 0.3 wt% sequentially. The pore diameter of the cryogels was counted, as shown in Fig. 3b–e, and the average pore diameters of GA/HD/MDP0, GA/HD/MDP1, GA/HD/MDP2, and GA/HD/MDP3 cryogels were 102.4, 78.0, 69.2, and 57.0 μm, respectively. It was mainly attributed to the increase in MDP nanoparticle concentration, which led to the reinforcement of hydrogen bonding in the cryogel network, and consequently resulted in the increase in cross-linking degree. In the compressed and fixed state, the macroporous structures of all cryogel groups showed a closed shape. However, after absorbing liquids, all of them quickly recovered the macroporous structure and exhibited no significant difference from the original state, indicating that compression fixation did not destroy the internal structure of the cryogels.

Fixed shape cryogels have the advantages of small size and easy storage. Meanwhile, it could swell after absorbing liquid and return to its original shape (Fig. 3f). To evaluate the swelling properties of GA/HD/MDP cryogels, the maximum compression ratio of the samples was tested first. As shown in Fig. 3g, the compression ratio of the cryogel gradually decreased as the MDP concentration increased, which was attributed to the reduced internal pore diameters. Subsequently, the swelling ratio and absorption time of GA/HD/MDP cryogels were assessed in deionized water and in whole blood, respectively. In deionized water (Fig. 3h, i), the time reaching balanced swelling of the cryogel accelerated and the swelling ratio decreased with the increasing of MDP concentration. It was primarily because of the increase in cross-linking density resulting in a diminished volume of the contained liquid and the enhanced mechanical properties. The extremely high swelling ratio of these cryogels was attributed to their rich macroporous structure and hydrophilic polymer composition, which can effectively absorb liquid many times their own weight. In blood (Fig. 3j, k), the swelling ratio of GA/HD/MDP1 decreased owing to the reduction in pore diameter. The increased swelling ratios of GA/HD/MDP2 and GA/HD/MDP3 as well as the decreased absorption time were associated with the formation of blood clots.

### Photothermal Effect and Antibacterial Ability of the Cryogels

**Fig. 4:**
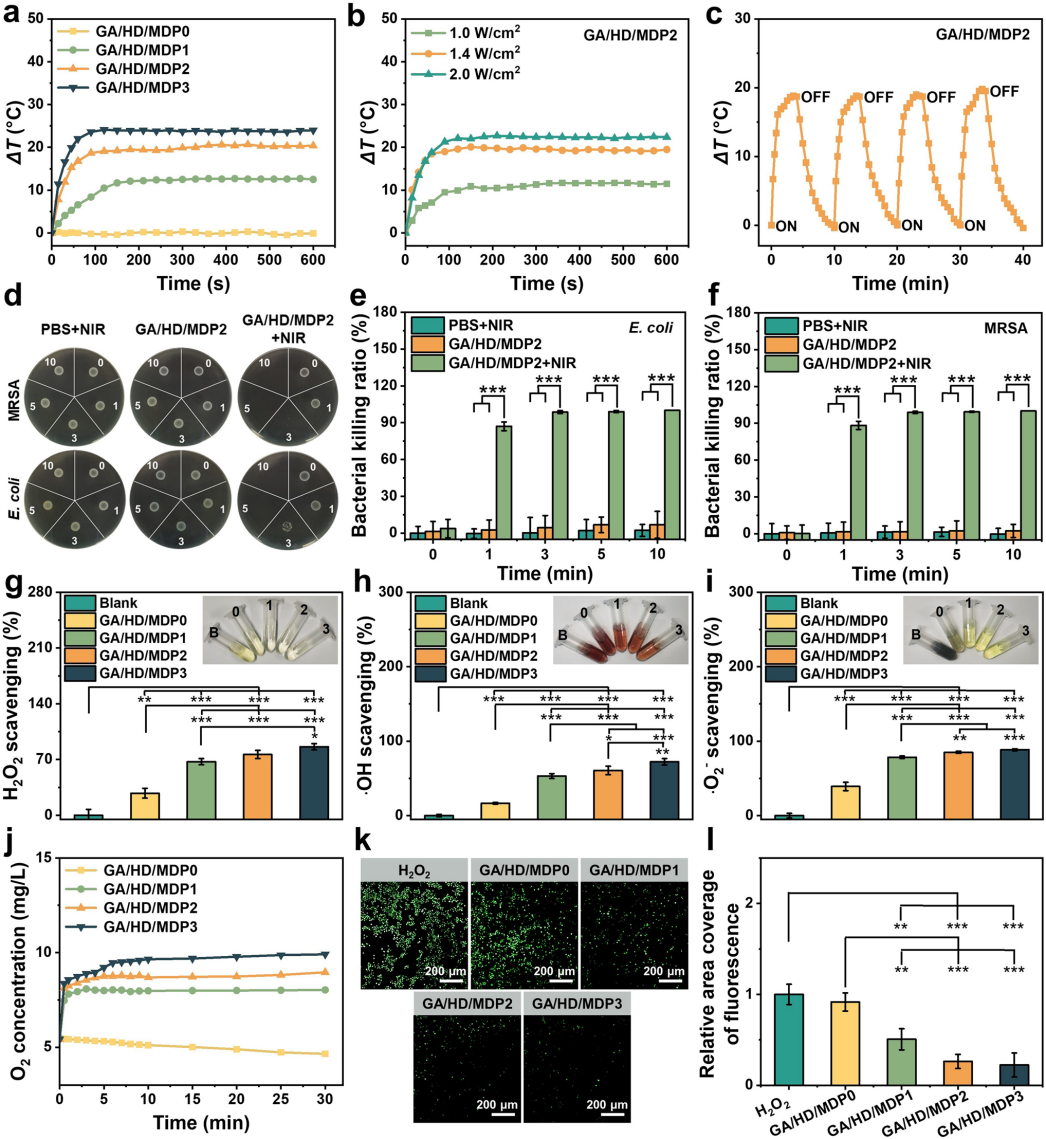
**a** Temperature variation curves of the GA/HD/MDP0, GA/HD/MDP1, GA/HD/MDP2, and GA/HD/MDP3 cryogels with a NIR light intensity of 1.4 W cm^−2^ for 10 min. **b** Temperature variation curves of the GA/HD/MDP2 cryogels with a NIR light intensity of 1.0, 1.4, 2.0 W cm^−2^ for 10 min. **c** *ΔT*-NIR light irradiation time curves of the GA/HD/MDP2 cryogel for four ON/OFF laser irradiation cycles. **d** Photographs of the survival *E. coli* and MRSA under the NIR irradiation. The photothermal killing ratio of GA/HD/MDP2 cryogel against **e** *E. coli* and **f** MRSA. Scavenging ratio of the cryogels to **g** H_2_O_2_, **h** ·OH, **i** ·O_2_^−^, the inset shows the color change of the indicator. B: blank, 0: GA/MD/MDP0, 1: GA/MD/MDP1, 2: GA/MD/MDP2, 3: GA/MD/MDP3; **j** Oxygen release curves of the cryogels. **k** Fluorescent images and **l** intensity counting of ROS expression in RAW 264.7 after treatment by 100 µM H_2_O_2_ and incubation with the cryogels for 12 h. (*P < 0.05, **P < 0.01, ***P < 0.001)

MnO_2_ and PDA have special optical properties that can absorb near-infrared light and convert it into thermal energy release [32, 33]. Thus, the photothermal converting efficiency and photothermal stability of the cryogels were evaluated. Firstly, the GA/HD/MDP cryogels were under NIR light for 10 min to test the photothermal converting property. As shown in Fig. 4a, the temperature of GA/HD/MDP0 cryogel had no change after NIR irradiation for 10 min. As the MDP nanoparticle concentration increased, the temperature rises of GA/HD/MDP1, GA/HD/MDP2, and GA/HD/MDP3 were about 12.5, 20.4, and 24 °C, respectively. Subsequently, the temperature rises of GA/HD/MDP2 cryogel under different power densities of NIR light were also tested (Fig. 4b). As shown in Fig. 4c, the *∆T* of GA/HD/MDP2 gradually increased from 11.5 to 19.5, and 22.4 °C under the power densities of 1.0, 1.4, and 2.0 W cm^−2^, indicating that it has tunable photothermal properties. Furthermore, the photothermal stability of GA/HD/MDP2 cryogel was further evaluated. After four cycles of NIR irradiation and natural cooling cycles, there was no significant difference in temperature increment (Fig. 4d). It was indicated that the GA/HD/MDP2 cryogel allowed repetitive photothermal antibacterial execution and guaranteed treatment efficacy.

Based on the photothermal properties of GA/HD/MDP cryogels, the photothermal bactericidal properties of GA/HD/MDP2 cryogel against *E. coli* and MRSA were further examined. As shown in Fig. 4d, bacterial colonies appeared in both the PBS + NIR group and the GA/HD/MDP2 cryogel without the NIR irradiation group. Whereas the GA/HD/MDP2 + NIR group showed a significant reduction in bacterial colonies after 3 min of NIR light irradiation, no bacterial colonies were observed when the irradiation time was extended to 5 and 10 min. The quantitative statistical results (Fig. 4e, f) showed that after NIR light irradiation for 1 min, the killing ratios of GA/HD/MDP2 cryogel against MRSA and *E. coli* were 88.2% and 87.0%, respectively. When the time was extended to 3 min, its killing ratios against both MRSA and *E. coli* reached more than 98.6%. The advantages of GA/HD/MDP2 cryogel in photothermal antibacterial was significantly better than those previously reported for photothermal hydrogel dressings [34, 35]. In summary, the above experimental results indicated that the GA/HD/MDP2 cryogel has good photothermal antibacterial properties.

### Antioxidant Property and Oxygen Release of the Cryogels

The self-regulation ability of the wound microenvironment in chronic diabetes is abnormal, and excessive production of ROS caused by oxidative stress prolongs the wound healing time. Dressings with ROS scavenging function can effectively improve the level of oxidative stress at the wound site. Therefore, the antioxidant properties of GA/HD/MDP cryogels were evaluated. The CAT-like activity of the cryogels was first tested. As shown in Fig. 4g, the solution gradually transitioned from yellow to colorless with the increase in MDP nanoparticles concentration, indicating that H_2_O_2_ was gradually removed. The quantitative results showed that the H_2_O_2_ scavenging ratios of GA/HD/MDP1, GA/HD/MDP2, and GA/HD/MDP3 were 67.3%, 76.4%, and 86.2%, respectively. It was mainly attributed to the MnO_2_ catalyzed H_2_O_2_ into water and oxygen. Whereas, the GA/HD/MDP0 cryogel without MDP nanoparticles showed a 27.7% scavenging ratio, which could be attributed to the diluted concentration of H_2_O_2_ in the solution [4]. Subsequently, the scavenging ratios of the cryogel toward ·OH and ·O_2_^−^ were also evaluated (Fig. 4h, i). The results were similar to the H_2_O_2_ scavenging, showing a gradual increase in scavenging ratios as the concentration of MDP nanoparticles increased. To verify that cryogels drive oxygen release through enzyme-like activity. The oxygen release capability of the GA/HD/MDP cryogel was further evaluated. As shown in Fig. 4j, GA/HD/MDP0 cryogel did not detect O_2_ release within 30 min, which confirmed that MDP is the main cause of oxygen release. In contrast, the cryogel loaded with MDP nanoparticles exhibited a sustained release of O_2_ within 30 min, and the O_2_ release gradually increasing with the increase in MDP concentration.

Excessive expression of ROS attacks intracellular proteins and DNA, leading to cell death and preventing wound repair. Based on the proven antioxidant capacity in vitro, RAW 264.7 was used to further evaluate the antioxidant properties of GA/HD/MDP cryogels on the cellular level. As shown in Fig. 4k, GA/HD/MDP0 cryogel exhibited massive ROS (green fluorescence) similar to the H_2_O_2_ group owing to the absence of effective antioxidant components. As the concentration of MDP nanoparticles increased, the ROS fluorescence in RAW 264.7 cells showed a decreasing trend (Fig. 4l). Specifically, the GA/HD/MDP2 and GA/HD/MDP3 cryogels showed an extremely significant difference (*P* < 0.001) from the H_2_O_2_ group, suggesting that they could effectively scavenge ROS to alleviate oxidative stress, which was mainly attributed to the antioxidant property of MnO_2_ nanozyme in the cryogels.

### Biocompatibility of the Cryogels

**Fig. 5:**
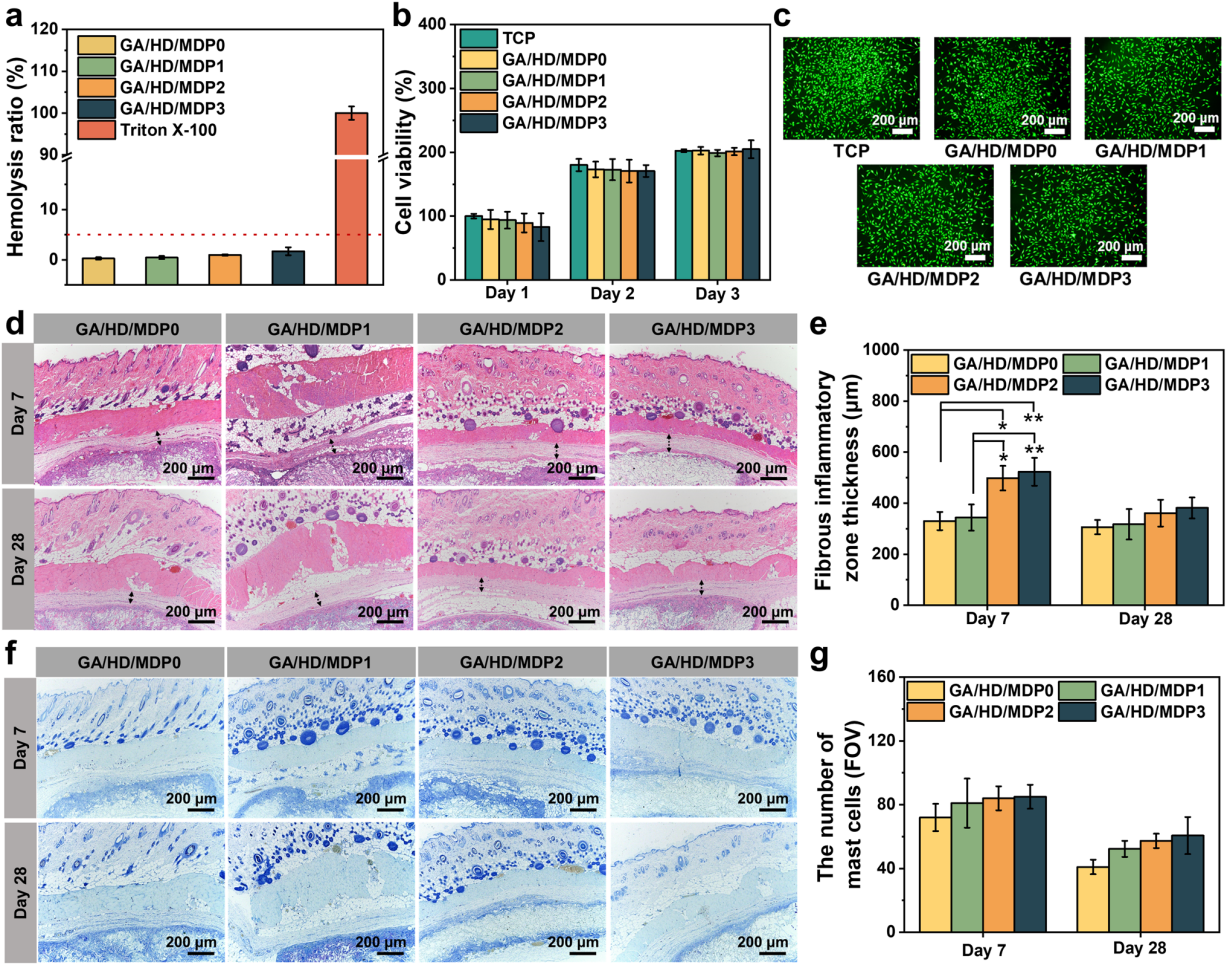
**a** In vitro hemolysis of GA/HD/MDP cryogels. **b** L929 cells viability. **c** Live/Dead staining of L929 cells after co-cultured with GA/HD/MDP cryogels for 24 h. Scale bar: 200 μm. **d** H&E and **f** TB staining results for day 7 and day 28. **e** Quantitative analysis of the thickness of the fibrous inflammatory zone. **g** Quantitative analysis of the mast cells in each field of view (FOV). (*P < 0.05, **P < 0.01, ***P < 0.001)

As hemostatic and repair materials are directly applied to the wound sites, it is important to avoid causing hemolysis, cytotoxicity, and severe immune reactions. Therefore, the biosafety of GA/HD/MDP cryogels was evaluated by hemocompatibility, cytocompatibility, and in vivo biocompatibility tests. Firstly, the cryogels were co-incubated with red blood cells for 1 h to estimate the hemolysis degree of the material. As shown in Fig. 5a, the supernatants of all cryogel groups showed a light pink color similar to the PBS group, while the Triton X-100 group showed a bright red color due to the complete rupture of the red blood cells. The hemolysis ratios of GA/HD/MDP0, GA/HD/MDP1, GA/HD/MDP2, and GA/HD/MDP3 cryogels were 0.26%, 0.44%, 0.95%, and 1.7%, respectively (Fig. 5a). These results indicated that the hemolysis ratio of GA/HD/MDP cryogels showed an increasing trend with the rising concentration of MDP nanoparticles. Nevertheless, all cryogels’ hemolysis ratios were lower than 5% (a safety threshold defined clinically), indicating that GA/HD/MDP cryogels have good hemocompatibility.

Subsequently, the cytotoxicity of GA/HD/MDP cryogels was evaluated by direct contact assay with L929 cells. Live/Dead staining images (Fig. 5b) showed that after the cells co-incubated with the cryogels for 24 h, the L929 cells in the GA/HD/MDP cryogel groups were similar to the TCP group, showing the characteristic spindle shape of fibroblasts (green fluorescence). As shown in Fig. 5c, the quantitative results indicated that after co-incubation for 24 h, the cell viabilities of GA/HD/MDP0, GA/HD/MDP1, GA/HD/MDP2, and GA/HD/MDP3 cryogels were 98.5%, 95.9%, 90.7%, and 85.6%, respectively, which were higher than 80%. Moreover, there was no statistically significant difference in cell viability between all the cryogel groups and the TCP group after co-incubation for 48 and 72 h (*P* > 0.05). The above test results demonstrated that the GA/HD/MDP cryogels have good cytocompatibility.

Furthermore, the in vivo biocompatibility of GA/HD/MDP cryogels was further evaluated by subcutaneous implantation. GA/HD/MDP0 without nanoparticles was used as the control group. The results of H&E staining (Fig. 5d) showed that after implantation for 1 week, all cryogel groups exhibited the acute inflammatory response, and the thickness of the fibrous inflammatory zone of GA/HD/MDP2 and GA/HD/MDP3 were significantly higher than that of GA/HD/MDP0 (*P* < 0.05). Specifically, neutrophils and macrophages showed a slight increasing trend, indicating that cryogel implantation induced a normal immune response in the body in the early stage. After implanting for 4 weeks, accompanied by the fibrous inflammatory zone thickness decreasing, the inflammatory response of all the cryogel groups was significantly reduced, the inflammatory cells at the implantation site almost disappeared, and there was no significant difference between each group (Fig. 5e). Additionally, TB staining (Fig. 5f) showed similar results to H&E. Mast cells play an important role in the early stages of allergic reactions and inflammation by releasing histamine and inflammatory mediators, leading to local inflammatory responses. The quantitative statistics (Fig. 5g) showed that the number of mast cells at the implantation site increased with the MDP concentration increasing after implantation for 1 week. After 4 weeks, the number of mast cells in all groups was significantly reduced, which indicated the weakening of the inflammatory response. The above results comprehensively indicated that the GA/HD/MDP cryogel has good biocompatibility.

### In Vitro Blood Clotting Capability of the Cryogels

**Fig. 6:**
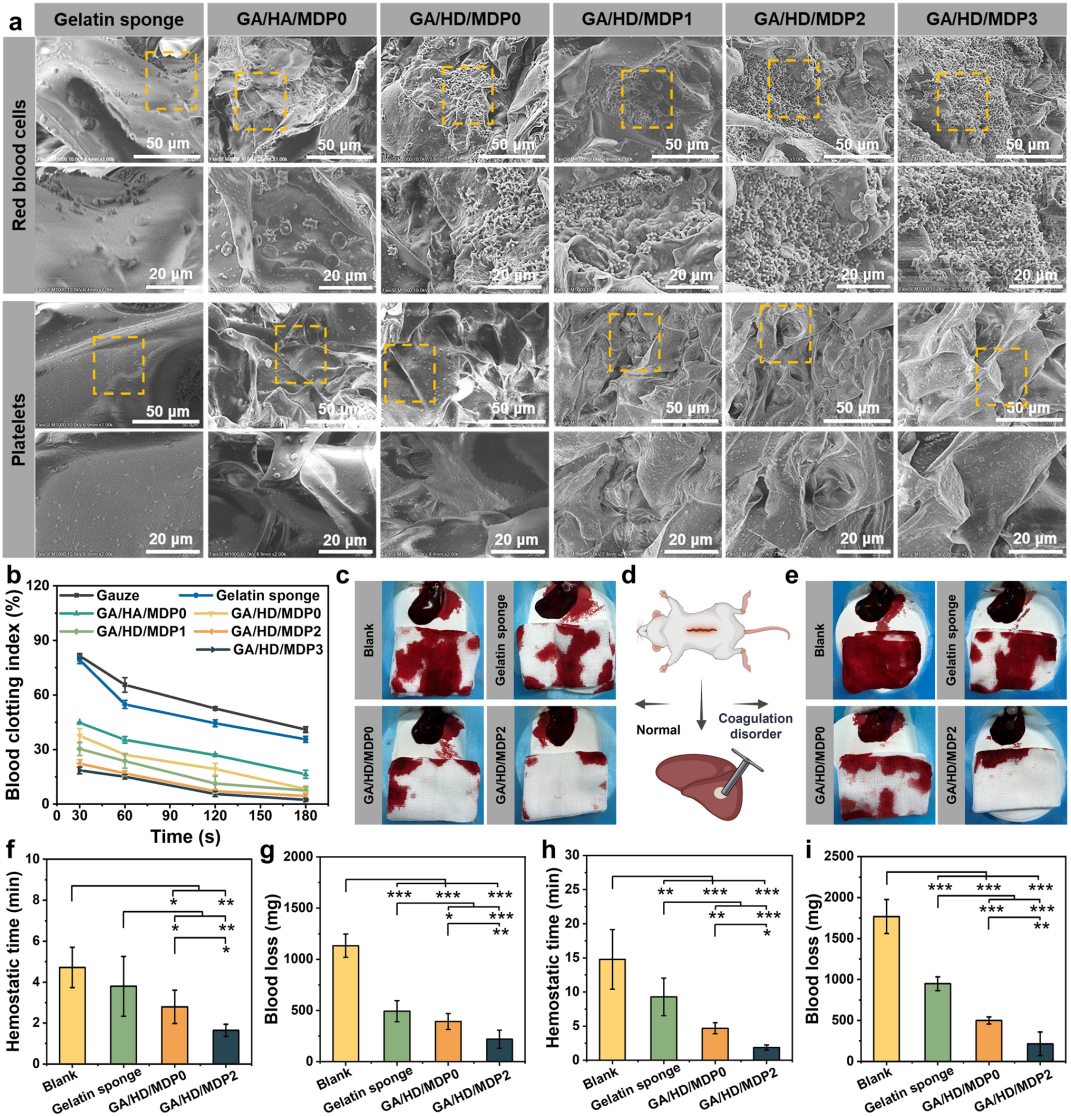
**a** SEM images of red blood cells and platelets adhering to the gelatin sponge, and GA/HD/MDP cryogels. **b** In vitro dynamic whole blood clotting test. The bloodstain photographs of **c** SD rat liver defect hemorrhage model and **e** coagulopathic SD rat liver defect hemorrhage model. **d** Schematic diagram. The **f**, **h** hemostatic time and **g, i** blood loss of SD rat and coagulopathic SD rat liver defect hemorrhage model, respectively. (*P < 0.05, **P < 0.01, ***P < 0.001)

The interconnected macroporous structure of the cryogel allows it to be compressed over 90% of its volume, and it can swell quickly by absorbing blood and achieve good hemostasis by concentrating blood. Thus, the hemostatic potential of GA/HD/MDP cryogels was estimated by red blood cells and platelets adhesion test using a commercial hemostatic material gelatin sponge as the control group. As shown in Fig. 6a, all the cryogels exhibited more red blood cell adhesion than gelatin sponges, and because of the presence of dodecyl hydrophobic chains, the red blood cell density was higher in the GA/HD/MDP0 group compared with the GA/HA/MDP0 group. As the increased concentration of MDP nanoparticles, besides the increasing number of adhered red blood cells, more red blood cells were activated from the discus shape with two sides concave to the irregular shape. Similar results were shown in the platelet adhesion assay. The GA/HD/MDP cryogel group with MDP exhibited more platelet adhesion than the gelatin sponge and GA/HA/MDP0 groups owing to the anchoring effect of the dodecyl and the chemical activation of PDA. Interestingly, platelets on the cryogel surface formed filopodia, a marker of their activation. As a result, GA/HD/MDP cryogels could effectively adhere to and activate red blood cells and platelets through physical and chemical synergy effects, thus promoting the formation of blood clots.

Furthermore, the in vitro clotting properties of GA/HD/MDP cryogels were quantitatively evaluated by the dynamic whole blood clotting assay. As shown in Fig. 6b, using gauze and gelatin sponge as the control group, the blood clotting index (BCI) of each group decreased as time increased. The BCI value of all the cryogel groups was significantly lower than that of the control group, which was mainly attributed to the macroporous structure of the cryogel, effectively absorbing and concentrating blood. There are also differences among the cryogel groups. Specifically, GA/HD/MDP0 always had a lower BCI than GA/HA/MDP0 cryogel, which was attributed to the dodecyl on HD which anchored red blood cells and platelets more efficiently. Moreover, as the MDP concentration increased, the BCI of the cryogel gradually decreased and showed a better blood clotting effect, which was related to the adhesion and activation effect of PDA. After incubating for 180 s, the BCI values of gauze and gelatin sponge were 40.9% and 35.7%, respectively. In contrast, the BCI value of GA/HA/MDP0 was significantly dropped to 16.4%, and the BCI values of GA/HD/MDP0, GA/HD/MDP1, GA/HD/MDP2, and GA/HD/MDP3 cryogels were 8.4%, 7.5%, 4.6%, and 2.4%, respectively, indicating that GA/HD/MDP cryogel has a better blood clotting effect.

### In Vivo Hemostatic Capability of the Cryogels

Patients with coagulopathy lack coagulation factors and platelet function is abnormal, so the wound site is susceptible to prolonged bleeding time and increased bleeding volume. Thus, a rat liver defect model and a drug-induced coagulopathy rat liver defect model were constructed (Fig. 6d), and the gelatin sponge was used as the control group. Meanwhile, GA/HD/MDP0 without MDP nanoparticles and GA/HD/MDP2 with good in vitro hemostatic properties were selected to evaluate the hemostatic properties of the cryogel. As shown in Fig. 6c, in the liver non-compressible hemorrhage model of normal rats, the blood loss in the blank and gelatin sponge groups was significantly higher than that in the two cryogel groups. Quantitative results showed (Fig. 6g) that the blood loss in the blank group showed extremely significant difference from the other three groups (*P* < 0.001), and the blood loss in the gelatin sponge group was about 100 mg higher than that in the GA/HD/MDP0 group and more than twice as much as that in the GA/HD/MDP2 group. It was attributed to the cryogels’ ability to swell faster and self-adapting the wound shape, resulting in a better filling effect. Moreover, the ability of these cryogels to absorb and concentrate blood was superior to the gelatin sponge. For the cryogel groups, GA/HD/MDP2 had less blood loss (220 mg) and shorter hemostatic time (1.6 min) compared with GA/HD/MDP0. It was mainly attributed to covalent and non-covalent interactions between catechol moieties of PDA and reactive residues of proteins as well as polysaccharides in blood cells, which makes the cryogel efficiently adhere and activate red blood cells and platelets, further accelerating the formation of blood clots.

As shown in Fig. 6e, in the liver non-compressible hemorrhage model of coagulopathic rats, the blood loss in the blank group was about 1769 mg, which was significantly higher than that in the blank group of normal rats (about 1133 mg), demonstrating the successful establishment of the model. The quantitative results (Fig. 6i) showed that there was a highly significant difference (*P* < 0.001) in blood loss between the blank group and three treatment groups, indicating that both gelatin sponge and cryogel exhibited hemostatic effects. However, the gelatin sponge still exhibited significantly higher blood loss (*P* < 0.001) and hemostatic time (*P* < 0.01) than the GA/HD/MDP0 cryogel group. It was related to the macroporous structure of the cryogel and the physically synergistic clotting effect of the dodecyl hydrophobic chains. Moreover, the GA/HD/MDP2 cryogel group still exhibited the lowest blood loss (215 mg) and the fastest hemostatic time (1.9 min) among all groups. It can be attributed to the four following effects. The first was the adhesion and activation of platelets by gelatin. The second was the expanding and concentrating effect of the cryogel. The third was the hydrophobic interaction between the dodecyl chains and the red blood cells and platelets. The fourth was PDA binding to protein and polysaccharide receptors on cell membranes that effectively adhere and activate blood contents. The above hemostatic strategies enable the cryogel to promote clot formation independent of coagulation factors and coagulation cascade reactions. In vivo clotting assays demonstrated the potential of GA/HD/MDP cryogels for application to non-compressible bleeding hemostasis in coagulopathy patients.

### In Vivo Wound Healing Performance of the Cryogels

**Fig. 7:**
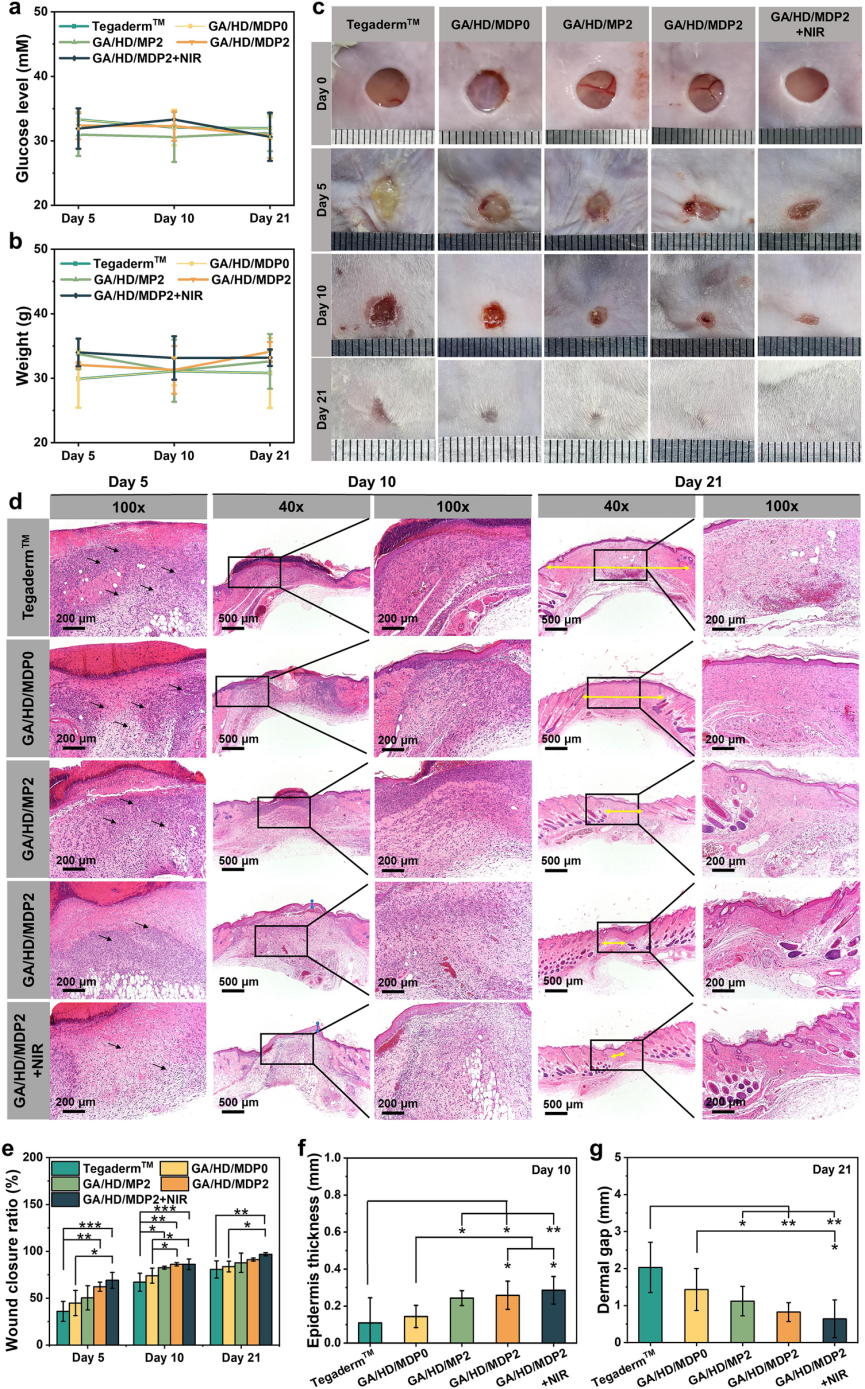
**a** Glucose level and **b** weight of mouse on day 5, 10, and 21. **c** Photographs of the wounds on day 0, 5, 10, and 21. **d** H&E staining of wound tissue sites on day 5, 10, and 21. **e** Wound closure ratio of the chronic mouse MRSA-infected wounds. **f** The quantitative data of wounds epidermis on day 10. **g** Wounds dermal gap on day 21. (*P < 0.05, **P < 0.01, ***P < 0.001)

Based on the above test results, GA/HD/MDP2 cryogel with good swelling properties, biocompatibility, antioxidant and photothermal antibacterial properties was selected to evaluate the healing effect of the cryogel by MRSA-infected diabetic full-thickness skin defect model. The commercial dressing Tegaderm™ film was used as the control group, and the GA/HD/MDP2 + NIR group was used as the experimental group. Meanwhile, to better illustrate the effect of different components in wound healing, GA/HD/MDP0 cryogel without MDP nanoparticles, GA/HD/MP2 cryogel without DFO, and GA/HD/MDP2 cryogel without the NIR assistance were used as the other three experimental groups, respectively. Type I diabetic mice were established by intraperitoneal injection of high-dose streptozotocin. The glucose and weight of mice were monitored throughout the wound healing process. As shown in Fig. 7a, b, the mice maintained high glucose levels from day 0 to day 21, and the mice showed no significant increase in weight, which exhibited the characteristics of type I diabetes, confirming the successful establishment of the mice diabetes model.

Figure 7c shows photographs of the wound healing on days 0, 5, 10, and 21. After 5 days, the GA/HD/MDP2 + NIR group killed numerous bacteria at the wound site because of timely and effective photothermal antibacterial treatment. The other four groups showed different degrees of bacterial infection, and the Tegaderm™ group had the most obvious yellow biofilm. The quantitative results (Fig. 7e) showed that after the 5th day of treatment, the wound closure ratios of all cryogel groups were higher than the Tegaderm™ group. Among them, the closure ratio of GA/HD/MDP0 group was about 8.8% higher than the Tegaderm™ group, which was mainly attributed to the ability of cryogel to absorb wound exudate, while the three-dimensional network composed of gelatin and hyaluronic acid provided a favorable ECM-like environment for tissue repair. With the introduction of MDP nanoparticles, MnO_2_ scavenged endogenous ROS while releasing O_2_, which alleviated the oxidative stress and hypoxic microenvironment at the wound site. Therefore, GA/HD/MP2 and GA/HD/MDP2 had higher wound closure ratios of 50.3% and 62.2%, respectively. With the assistance of NIR, the bacterial infection degree at the wound site was significantly weakened, resulting in the reduction in inflammatory response. As a result, the GA/HD/MDP2 + NIR group had the highest wound closure ratio (69%). On day 10 of wound healing, all groups showed further wound closure, while the Tegaderm™ group still exhibited the lowest wound closure ratio compared with the other cryogel groups. Among the experimental groups, the closure ratios of the GA/HD/MP2 and GA/HA/MDP2 groups had no significant difference with the GA/HD/MDP2 + NIR group, indicating that photothermal antibacterial property play a dominant role in the early stage of wound healing, and further wound healing is relying on the antioxidant property of MnO_2_ to accelerate the inflammatory stage ending. On day 21, the wounds in the GA/HD/MDP2 + NIR group were almost completely closed, which was similar to normal skin. Meanwhile, there were still significant differences between the Tegaderm™ group (*P* < 0.01) and the GA/HD/MDP0 group (*P* < 0.05). The above results indicated that GA/HD/MDP0 cryogel based on gelatin and hyaluronic acid was significantly superior to the Tegaderm™ dressing in bacterial-infected diabetic wound healing. GA/HD/MP2 and GA/HD/MDP2 cryogels, which catalyzed the decomposition of endogenous ROS and generated O_2_, could promote wound closure. With the assistance of NIR, the highly effective antibacterial effect in the early stage and the antioxidant properties in the middle and late stages of the cryogel have a dual effect in reducing the oxidative stress at the wound site, which can accelerate wound healing more effectively.

To further illustrate the healing effect of the skin tissue, hematoxylin and eosin (H&E) staining was carried out on the regenerated tissues at the wound site at different time points of healing to evaluate inflammation and regeneration at the wound site. As shown in Fig. 7d, on day 5, all groups except the GA/HD/MDP2 + NIR group exhibited severe inflammatory infiltration (black arrow), which was caused by bacterial infection. On day 10, the Tegaderm™ group still exhibited numerous inflammatory cells. In contrast, the GA/HD/MP2 and GA/HD/MDP2 cryogel groups showed significantly reduced inflammatory response, which was attributed to the antioxidant properties of the cryogel that improved the microenvironment of the wound. Meanwhile, the GA/HD/MDP2 + NIR group still showed the least inflammatory cells, and the GA/HD/MP2, GA/HD/MDP2 and GA/HD/MDP2 + NIR groups presented intact epidermal structures. Quantitative statistical results (Fig. 7f) showed that the epidermal thickness of the GA/HD/MDP0 group was slightly higher than that of the Tegaderm™ group on day 10, but significantly lower than the other three cryogel groups (*P* < 0.05). On day 21, the Tegaderm™ and GA/HD/MDP0 groups also showed intact epidermis. The dermal gap reflects the remodeling level of regenerated tissue and is an important indicator of skin function reconstruction (yellow arrow) [36, 37]. The dermal gap in the Tegaderm™ group had significant differences compared with the GA/HD/MP2 group (*P* < 0.05) and highly significant differences compared with the GA/HD/MDP2 and GA/HD/MDP2 + NIR groups (*P* < 0.01) (Fig. 7g). Furthermore, H&E staining showed that the GA/HD/MDP2 + NIR group had more skin appendages and exhibited better healing quality. It was mainly because the GA/HD/MDP2 cryogel effectively killed bacteria with NIR synergism, and the MnO_2_ in the cryogel catalyzed the decomposition of endogenous ROS producing O_2_, which effectively regulated the level of oxidative stress and hypoxia at the wound site, thus promoting wound healing of infected diabetes wounds.

### Collagen Deposition, CD80, CD206, and VEGF of the Regenerated Tissue

**Fig. 8:**
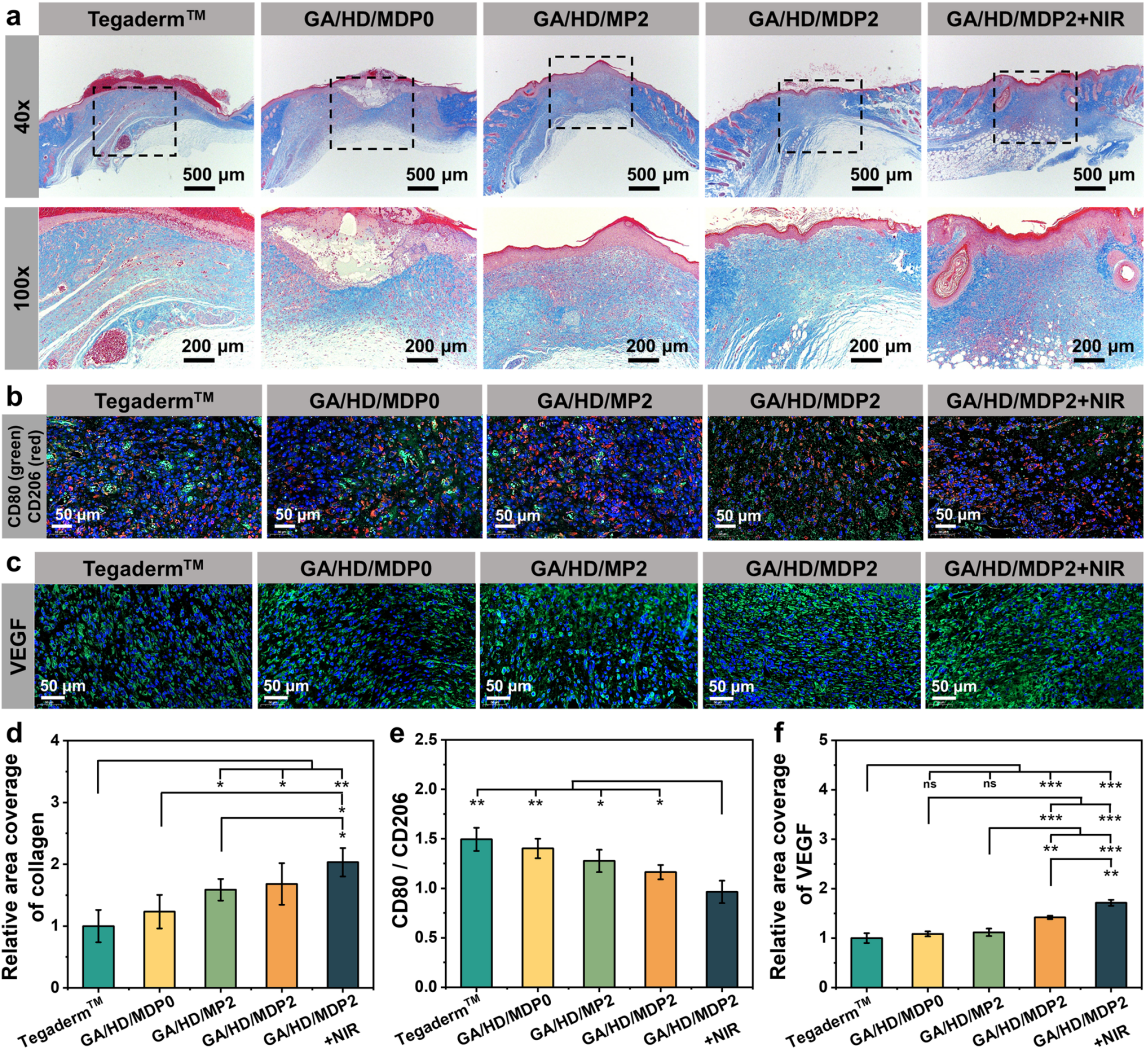
**a** Masson staining of wounds on day 10. Immunofluorescence staining of **b** CD80 & CD206 on day 5 and **c** VEGF on day 10. Relative fluorescence intensity of **d** collagen on day 10, **e** CD80/CD206 on day 5, and **f** VEFG on day 10. *P < 0.05, **P < 0.01, ***P < 0.001

In the middle and late stages of wound healing, more fibroblasts promote the synthesis of stable ECM, which produces a denser collagen skeleton [38]. Therefore, the level of collagen deposition at the wound sites was evaluated by Masson staining. As shown in Fig. 8a, on day 10, the collagen in the Tegaderm™ group showed a loose reticulated fiber structure and the level of collagen deposition was lower than that of all the cryogel groups. Among all cryogel groups, the GA/HD/MDP2 + NIR group exhibited a denser and more regular collagen fiber structure. Its collagen deposition levels were 1.7, 1.3, and 1.2 times higher than those of GA/HD/MDP0, GA/HD/MP2, and GA/HD/MDP2 groups, respectively (Fig. 8d). It was mainly attributed to the dual effect of NIR antibacterial and antioxidant of GA/HD/MDP2 cryogel, which reduced the oxidative stress at the wound site [19, 39, 40]. The increase in O_2_ concentration provided energy for cell activities, which in turn promoted collagen deposition [41]. Furthermore, gelatin and hyaluronic acid in the cryogel can promote cell adhesion, proliferation, and migration, effectively promoting collagen deposition at the wound site [42–44].

Because of excessive inflammation, wounds typically fail to enter the proliferation stage and remain in the inflammation stage for a long time [45]. Therefore, on day 5, immunofluorescence staining of the regenerated tissue was performed using CD80 (a surface marker for M1-type macrophages) and CD206 (a surface marker for M2-type macrophages) to monitor the level of inflammation at the wound site. As shown in Fig. 8b, significant green fluorescence (CD80) could be observed in Tegaderm™ and GA/HD/MDP0 groups, indicating the dominance of M1-type macrophages at the wound site, while green fluorescence was significantly reduced in the other three cryogel groups. The CD80/CD206 results (Fig. 8e) showed that the ratio of GA/HD/MDP0 cryogel was slightly lower than that of the Tegaderm™ group. However, the expression of pro-inflammatory macrophages was higher than anti-inflammatory macrophages in both groups, and the wounds were still in a severe inflammation stage. In the GA/HD/MP2 and GA/HD/MDP2 groups, the expression of CD80 and CD206 were comparable. Nevertheless, the expression of CD80 was lower than that of CD206 in the GA/HD/MDP2 + NIR group, suggesting a tendency for macrophages to shift from the M1 to the M2 type. It indicated that the cryogel with photothermal antibacterial and antioxidant properties could effectively reduce the inflammatory response and accelerate the transition from inflammation to the proliferation stage [32].

On the other hand, angiogenesis is crucial for wound healing as it provides the tissues with essential nutrients and metabolic pathways [46]. Thus, the expression of VEGF was detected by immunofluorescence staining to evaluate the angiogenesis in the neonatal skin tissues on day 10. As shown in Fig. 8c, the green fluorescence levels were significantly higher in the GA/HD/MDP2 and GA/HD/MDP2 + NIR groups. The quantitative results (Fig. 8f) showed that the VEGF expression of GA/HD/MDP0 and GA/HD/MP2 cryogels was slightly higher than that of Tegaderm™ film. This was mainly because the cryogel effectively absorbed tissue exudate which reduced bacterial colonization, and the antioxidant properties of MnO_2_ assisted in shortening the inflammation stage and accelerating the healing process [47, 48]. In addition, the expression of VEGF in GA/HD/MDP2 and GA/HD/MDP2 + NIR cryogel were significantly higher than that in the other three groups (*P* < 0.01), which was mainly attributed to the release of DFO. It could effectively promote vascular regeneration, which was more conducive to promoting the three-dimensional assembly of the vascular network and significantly improving the quality of wound healing [49, 50].

Overall, GA/HD/MDP cryogel has a good effect on the hemostasis of coagulopathy and on the repair of diabetic wounds, and these are mainly attributed to the following three points. Firstly, gelatin-based cryogel could promote the adhesion of blood cells and tissue cells [51, 52]. Meanwhile, its liquid-triggered shape recovery function could stop bleeding through expansion and concentration mechanisms [53], and its interconnected macroporous structures could absorb tissue exudates during the repair stage [6]. Secondly, the dodecyl side chain can effectively capture red blood cells and platelets through hydrophobic interaction. At the same time, the catechol structure of PDA provides a chemical binding site, which can induce the aggregation and activation of blood cells [10, 12]. The combination of physical and chemical effects makes the cryogel have the advantage of hemostasis independent of coagulation factors and coagulation cascade reactions. Finally, the introduction of MDP nanoparticles endows the cryogels with good photothermal antibacterial properties, MnO_2_-dependent ROS scavenging and oxygen release properties, and DFO sustained release angiogenic properties. This cryogel can effectively kill bacteria, improve wound microenvironment, promote tissue and vascular regeneration, and thus show a higher wound closure ratio and better functional repair than Tegaderm™ film.

## Conclusion

In conclusion, a series of GA/HD/MDP cryogels with physical and chemical synergistic hemostasis, photothermal antibacterial, ROS scavenging, oxygen release, and angiogenic properties were successfully prepared. The mechanical strength and absorption capability of the cryogels can be adjusted based on the concentration of MDP nanoparticles. Its good biocompatibility was demonstrated by co-culture with blood and L929 cells, as well as in vivo subcutaneous implantation tests. In vitro tests showed that the cryogel had good photothermal antibacterial, ROS scavenging, and oxygen release properties. In addition, in vitro and in vivo hemostatic tests showed that GA/HD/MDP cryogel had a better clotting effect than gelatin sponge under the combined effect of dodecyl and polydopamine. In vivo MRSA-infected diabetic wound healing test demonstrated that GA/HD/MDP cryogel could decrease wound inflammation and oxidative stress, alleviate the hypoxic environment, promote collagen deposition, and induce vascular regeneration. In conclusion, GA/HD/MDP cryogel has potential application in non-compressible hemostasis of patients with coagulopathy and infected wound healing of diabetic patients.

## Supplementary Information

Below is the link to the electronic supplementary material.

Supplementary file1 (DOCX 154 KB)
