# Supplementary material for: All-in-One: A Multifunctional Composite Biomimetic Cryogel for Coagulation Disorder Hemostasis and Infected Diabetic Wound Healing
Source: Nanomicro Lett. 2025 Mar 3;17:171. doi: 10.1007/s40820-024-01603-1 (PMC11872855; doi:10.1007/s40820-024-01603-1)
Supplement: Supplementary file 1 — Supplementary file1 (DOCX 154 KB) [file 40820_2024_1603_MOESM1_ESM.docx]

Supporting Information for

**All-In-One: A Multifunctional Composite Biomimetic Cryogel for Coagulation Disorder Hemostasis and Infected Diabetic Wound Healing**

Jiaxin Wang^1,2, #^, Yutong Yang^2, #,^ *, Huiru Xu^2^, Shengfei Haung^2^, Baolin Guo^1, 2,^*, Juan Hu^1,^ *

^1^ Department of Otorhinolaryngology, Head and Neck Surgery, the Second Affiliated Hospital of Xi'an Jiaotong University, Xi'an 710004, P. R. China

^2^ State Key Laboratory for Mechanical Behavior of Materials, and Frontier Institute of Science and Technology, and Key Laboratory of Shaanxi Province for Craniofacial Precision Medicine Research, College of Stomatology, Xi’an Jiaotong University, Xi’an, 710049, P. R. China

^#^ Jiaxin Wang and Yutong Yang contributed equally to this work.

*****Corresponding authors. E-mail: [ytyang0718@xjtu.edu.cn](mailto:ytyang0718@xjtu.edu.cn) (Yutong Yang); [baoling@mail.xjtu.edu.cn](mailto:baoling@mail.xjtu.edu.cn) (Baolin Guo); [hjdoc@163.com](mailto:hjdoc@163.com) (Juan Hu)

**S1 Materials and methods**

**S1.1 Materials**

Gelatin (Gel, from porcine skin), adipic acid dihydrazide (ADH), 1-hydroxybenzotriazole (HOBt, 97.5%), N-hydroxysuccinimide (NHS), 1-ethyl-3-(3-(dimethylamino) propyl) carbodiimide hydrochloride (EDC·HCl, 98%), hyaluronic acid (HA, MW = 2-4 kDa), and dodecylamine (DDA) were obtained from Sigma-Aldrich. Sodium hydroxide (NaOH, 99%), hydrochloric acid (HCl, 99%), sodium chloride (NaCl), sodium carbonate (Na_2_CO_3_), hydrogen peroxide (H_2_O_2_, 30 wt%), and potassium permanganate (KMnO_4_) were obtained from Sinopharm. Dopamine hydrochloride (DA) was obtained from Energy Chemical. Treaethyl orthosilicate (TEOS), rivaroxaban, and deferoxamine (DFO) were obtained from Aladdin. Streptozocin (STZ) was obtained from Meryer.

**S1.2 Synthesis of adipic acid dihydrazide modified gelatin (GA)**

The grafting of hydrazide onto gel was carried out according to previous report [S1]. 1 g gelatin was dissolved in 100 mL deionized water, and 6 g ADH was added with stirring. Then, 1.83 g HOBt and 2.79 g EDC were dissolved in the 6.5 mL DMSO/H_2_O (1:1 v/v) mixture, and added to Gel/ADH mixture with stirring. The pH of the solution was adjusted to 5 and the amidation reaction was performed at room temperature for 24 h. Subsequently, NaCl was added to produce a 5% w/v solution and the Gel-ADH was precipitated in ethanol. After dissolution in deionized water, the precipitate was dialyzed for another 5 days to remove the salt. Finally, the solution was lyophilized to obtain a dried GA foam.

**S1.3 Synthesis of dodecylamine modified hyaluronic acid (HD)**

The amphiphilic polymer HA-DA was synthesized via the amidation reaction between HA and DA [S2]. Briefly, 388 mg (0.5 mmol) HA was dissolved in 30 mL distilled water and EDC·HCl (192 mg, 1 mmol)/NHS (115 mg, 1 mmol) were added to activate the carboxyl groups of HA for 1  h at 45  °C. 11.5 mg (0.0625 mmol) DA in 20 mL ethanol was then added into the HA solution, and the reaction proceed at 45 °C for 24 h. The products were dialyzed against distilled water for 5 days to remove byproducts, followed by lyophilizing to obtain HD.

**S1.4 Synthesis of MnO_2_@DFO@PDA nanoparticle (MDP)**

Firstly, SiO_2_ nanoparticles were synthesized according to previously reported method [S3]. In brief, ethanol (280 mL), deionized water (40 mL), and ammonia water (10 mL) were added into a round-bottomed flask and magnetically stirred at 50 °C for 5 min. TEOS (10 mL) was then added dropwise to the above mixture, stirred for 2 h, and then centrifuged at 12000 rpm for 5 min to obtain mesoporous SiO_2_ nanoparticles.

Secondly, an aqueous solution of KMnO_4_ (6 g) was dropwise added into the suspension of SiO_2_ (800 mg, 100 mL) under ultrasonication. Ultrasonication was continued for another 1 h after the KMnO_4_ was completely added, and the solution was stirred overnight at room temperature. The reactant was centrifuged at 12000 rpm for 5 min, and the supernatant was removed to obtain SiO_2_@MnO_2_ nanoparticles. These nanoparticles were then dispersed into sodium carbonate solution (2 M, 200 mL) and stirred at 60 ℃ overnight, after which the reactant was centrifuged at 12000 rpm for 5 min. The precipitate was washed with deionized water to obtain h-MnO_2_ [S4].

Then, for DFO loading, h-MnO_2_ NPs (10 mg) were added to 10 mL of phosphate buffered saline (PBS, pH 7.4) containing DFO (10 mg) and stirred for 12 h in darkness.

The solution was then centrifuged to remove the remaining DFO and obtain MnO_2_@DFO.

Finally, the MnO_2_@DFO NPs (10 mg) were dispersed in 10 mL of Tris buffer (pH 8.5, 1×10^−2^ M) containing dopamine hydrochloride (10 mg) for 6 h at room temperature in darkness and stirred to obtain MnO_2_@DFO@PDA. The final product (MDP NPs) was centrifuged and lyophilized for 48 h.

**S1.5 Synthesis of GA/HD/MDP cryogel**

Firstly, GA and HD were dissolved in deionized water at 37 °C to form 6.25 wt% and 1.25 wt% solution, respectively. Furthermore, MDP nanoparticles were added to deionized water to form 10, 20, 30 mg/mL dispersions, which were uniformly dispersed under ultrasound. Then, GA, HD, MDP, EDC (75 mg/mL) and NHS (45 mg/mL) solutions were pre-chilled in an ice-water mixture for 2 h. Add 400 µL GA solution, 400 µL HD solution, and 100 µL MDP dispersions to the centrifuge tube. After sufficient shaking, add 50 µL EDC, 50 µL NHS and oscillate sufficiently. Then, immediately immerse in iced ethanol (-20 °C) for 24 h, and later it was transferred to a -7 °C refrigerator for 24 h. Finally, the cryogel was immersed in deionized water for 3 days to remove unreacted EDC and NHS. The cryogel was named GA/HD/MDP-X, X represents the concentration of MDP nanoparticles (mg/mL).

**S1.6 Characterization**

The synthesized Gel-ADH (GA) were dissolved in deuteroxide. The ^1^H nuclear magnetic resonance (^1^H NMR) of GA were recorded at 25 ℃.

Fourier transform infrared spectroscopy (FT-IR) of freeze-dried GA, HD, and GA/HD/MDP cryogel were recorded in the range of 4000-450 cm^-1^ by employing a Bruker ALPHA II FT-IR spectrometer.

The morphologies of the freeze-dried cryogels were observed using a field emission scanning electron microscope (FEI Quanta FEG 250). Before observation, the surface of the cryogels was sprayed with a gold layer. The average pore sizes of the cryogels were measured and calculated using Image J software based on the SEM photographs.

For the maximum compression strain of the cryogels, the freeze-dried cryogels were processed into a cylindrical shape (with original length of 5 mm as L_1_). Then, the lyophilized cryogels were compressed as much as possible and keep this state for 5 min. The length of shape-fixed cryogels were recorded as L_2_. Each group was repeated for 5 times. The maximum compression strain was calculated as follows:

Maximum compression strain = (L_1_ - L_2_) / L_1_ × 100%

For the equilibrium swelling ratio test of the cryogels, the cryogels (cylindrical shape with a diameter of 8 mm and height of 5 mm) were equilibrated in deionized water (or blood) at room temperature (25 ℃) and then weighed (W_w_). Following that, they were sucked dry by the filter paper (or freeze-dried) and weighed again (W_d_). Each group was repeated for 5 times. The swelling ratio (SR) was calculated as follows:

SR = (W_w_ -W_d_)/W_d_ × 100%

For the recovery time of the cryogels, the cryogels (cylindrical shape with a diameter of 8 mm and height of 5 mm) were sucked dry by the filter paper. Then the samples were soaked in water, and recovery time was measured. The test was cycled for five times.

**S1.7 DFO release test of GA/HD/MDP cryogels**

To follow the kinetics of DFO release, the cryogel sample (50 mg) were individually placed in 3 mL PBS (pH value = 7.4) release medium at 37 ℃ under stirring (100 rpm). The release vessels were all well-sealed to prevent medium evaporation. At pre-determined intervals, 1 mL samples were taken from the PBS medium. Then, 1 mL fresh PBS was added into the release medium to maintain the constant volume. The ferric chloride solution (3 mM) was mixed with the collected supernatant (1:1 v/v). After 30 min of reaction, the absorbance was read at 485 nm wavelength using a microplate reader (Molecular Devices) to calculate the release of DFO [S5]. Each experiment was done three times.

**S1.8 Mechanical performance**

The mechanical performance of the cryogels was tested by uniaxial and cyclic compression test using TA rheometer (DHR-2) at room temperature [S6]. The cryogels were fabricated as cylindrical shapes with a diameter of 8 mm and height of 5 mm. The freeze-dried cryogels were firstly swollen in DI water, and then the compression test of the wet cryogels was conducted at a strain speed of 50 µm/s. A drop of water was added around the wet cryogels sample on the platform before the test, and then 80% compression strain was employed to conduct the uniaxial compression test. Meanwhile, the fatigue resistance of the cryogels was measured by cyclic compression. The compression strain was firstly performed up to 80% strain and then released to 0% strain by using constant compression and release strain rate, which was repeated for 10 times.

**S1.9 Photothermal performance**

To test the cryogels’ photothermal property, the swollen cryogels were cut into disks (a diameter of 8 mm and a height of 5 mm) and then exposed to a NIR laser (MDL-III-808 nm-1000 mW, Changchun New Industries Optoelectronics Tech Co., Ltd.) at power density varying from 1.0 to 1.4, and 2.0 W/cm^2^ for 10 min, respectively. To study the photothermal stability of the cryogel, GA/HD/MDP was irradiated in four repeated cycles of 5 min irradiation ON and 5 min OFF. The heat maps and temperature profiles of the cryogels were recorded using an infrared (IR) thermal camera.

**S1.10 Photothermal antibacterial performance**

The GA/HD/MDP disks (with a diameter of 8 mm and height of 5 mm) were sterilized by immersing them in 75% alcohol and then equilibrated using sterilized PBS. 10 µL of *E. coli* and MRSA bacterial suspension in sterilized PBS (10^8^ CFU mL^-1^) was added onto the surface of the cryogels. After that, the samples were exposed to NIR laser light (808 nm, 1.4 W/cm^2^) for different time intervals from 0 to 1, 3, 5 and 10 min, respectively. 10 µL of bacterial suspension (10^8^ CFU mL^-1^) suspended in 990 µL of PBS was used as a negative control, which was also exposed to NIR laser light (808 nm, 1.4 W/cm^2^). 990 µL of sterilized PBS was introduced into each well to re-suspend any bacterial survivor. Then, 10 µL of the above resuspension was added onto agar plate, the colony-forming units on the agar plate were calculated after cultured for 12 h at 37 ℃. The antibacterial efficiency was expressed as antibacterial ratio using the following equation:

Bacterial killing ratio = ((bacterial count of control - survivor count on cryogel) / bacterial count of control) × 100%.

**S1.11 Antioxidant performance**

The antioxidant abilities of GA/HD/MDP cryogels were tested by scavenging efficiency for H_2_O_2_, ·OH, ·O_2_^-^, and intracellular ROS scavenging. H_2_O_2_ scavenging ability was evaluated according to the publication [S7]. 300 μL GA/HD/MDP cryogel samples were added to 1 mL PBS buffer containing H_2_O_2_ (2 mM) and incubated in the shaker at 37 °C for 4 h. 100 μL of supernatant was collected and mixed with 200 μL of titanium sulfate solution (1.33 mL of 24 % Ti(SO_4_)_2_ and 8.33 mL of H_2_SO_4_ dissolved in 50 mL deionized water) for 30 min, and then the 200 μL supernatant was introduced into a 96-well plate. The absorbance was read at 405 nm wavelength using a microplate reader (Molecular Devices). The scavenging ratio of H_2_O_2_ was calculated as follows:

H_2_O_2_ scavenging (%) = (A_m_ - A_n_) / A_m_ × 100%, Where A_m_ and A_n_ represent the absorbance values of the blank and the GA/HD/MDP cryogel, respectively.

·OH scavenging ability was evaluated according to the publication [S7]. The solution containing H_2_O_2_ (5 mM) and Fe^2+^ (2 mM) was incubated at 37 °C for 10 min to produce ·OH. Subsequently, 300 μL of GA/HD/MDP cryogel sample was added into 1 mL of the above solution and incubated at 37 °C for 30 min. Then 900 μL of the supernatant was mixed with 100 μL of salicylic acid (SA) solution (15 mM) for 30 min. Thus, ·OH scavenging activity of cryogels can be determined by detecting the 2, 3-dihydroxybenzoic acid with an absorption peak at 510 nm using a microplate reader (Molecular Devices). The scavenging ratio of ·OH was calculated as follows:

·OH scavenging (%) = (A_b_ - A_c_) / A_b_ × 100%,

Where A_b_ and A_c_ represent the absorbance values of the blank and the GA/HD/MDP cryogel, respectively.

·O_2_^-^ scavenging ability was determined by calculating the inhibition ratio of the photoreduction of NBT [S8]. Briefly, 300 μL GA/HD/MDP cryogel sample was added into 1 mL PBS buffer (pH 7.4) containing riboflavin (20 µM), methionine (12.5 mM) and NBT (75 µM), and treated with a constant light intensity for 30 min. Then, 200 μL of the supernatant was collected, and the absorbance was read at 560 nm wavelength using a microplate reader (Molecular Devices). The ·O_2_^-^ scavenging was calculated using equation:

·O_2_^-^ scavenging (%) = [(A_1_ - A_P_) - (A_0_ - A_p_) / (A_1_ - A_p_)] × 100%,

Where A_0_, A_p_, and A_1_ represent the absorbance values of the GA/HD/MDP cryogel, PBS, and positive control, respectively.

The intracellular ROS scavenging test was evaluated according to the publication [9]. Briefly, RAW 264.7 cells in a 48-well plate (3×10^4^ cells/well) were incubated with GA/HD/MDP for 24 h. Next, 500 µM H_2_O_2_ was added to the culture media to induce oxidative stress for 1 h. Then, the media and samples were aspirated, and the cells were washed carefully with PBS for 2 times. The cells were incubated with 10 µM DCFH-DA solution at 37 °C for 1 h in dark. The fluorescence intensity of DCFH-DA was read using a microplate reader (Ex. 495 nm; Em. 530 nm) and the fluorescence images were observed by confocal microscope.

**S1.12 Oxygen production performance**

The 10 mg GA/HD/MDP cryogel was placed in 5 mL of deionized water containing H_2_O_2_ (200 μM), and the concentration of O_2_ was recorded using a portable dissolved oxygen analyzer (JPBJ-608, Shanghai Leici) at the pre-set time.

**S1.13 Biocompatibility performance**

The biosafety of GA/HD/MDP cryogels was evaluated by hemocompatibility, cytocompatibility, and *in vivo* biocompatibility tests [S8].

For hemocompatibility test, erythrocytes were separated from the mouse blood by centrifugation at 116×g for 10 min. The obtained erythrocytes were washed three times with PBS and then diluted to a concentration of 5% (v/v). Then, the lyophilized cryogels were dispersed to form dispersion liquids with the concentration of 10 mg/mL. 0.5 mL of the dispersion liquid was added into 2 mL tube, and then gently mixed with 500 µL of erythrocyte suspension (5% (v/v)). After incubated at 37 ℃ for 1 h, the tubes were centrifuged (at 116×g) for 10 min. The obtained supernatants were introduced into 96-well microplate. The absorbance of the solution was read at 540 nm by a microplate reader (Molecular Devices). 0.1% Triton X-100 was used as the positive control while PBS was used as the negative control. Each group was repeated for three times. The hemolysis ratio was calculated as follows:

Hemolysis ratio (%) = [(A_c_ - A_b_) / (A_t_ - A_b_)] × 100%,

Where A_c_, A_t_, and A_b_ represent the absorbance values of the GA/HD/MDP cryogel, positive control, and PBS, respectively.

The cytotoxicity of the cryogels was evaluated by a contacting test method. For the contacting test method, the freeze-dried cryogels were firstly cut into disks with diameter of 8 mm and height of 1 mm, and then sterilized by immersing them into 75% alcohol. The complete growth medium was Dulbecco’s modified eagle medium (DMEM) (Gibco) supplemented with 10% fetal bovine serum (Gibco), 1.0 × 10^5^ U/L penicillin (Hyclone) and 100 mg/L streptomycin (Hyclone). L929 cells were seeded into 48-well plate with cell density of 7500 cells/well. After cultivated for one day, the cryogels disks equilibrated in the culture medium were added into each well and kept the culture medium level slightly lower than the cryogels upper surface to ensure the fine contact between cell and cryogels. After cultured for 24 h, the cell viability under the cryogels was tested by Live/Dead Viability/Cytotoxicity Kit assay. After cultured for 1 day, 2 days and 3 days, respectively, the cell viability under the cryogels were tested by alamarBlue^®^ assay. 20 µL of alamarBlue^®^ reagent in 200 µL culture medium was added into each well after removing the cryogels disks and medium. After incubated at 37 ℃ for 4 h, 100 µL of the medium in each well was transferred to a 96-well black plate (Costar). Fluorescence was read by a microplate reader (Ex. 560 nm; Em. 600 nm). Tests were repeated four times for each group. Cell adhesion and viability were observed under an inverted fluorescence microscope (IX53, Olympus).

For *in vivo* biocompatibility, all the samples were cut into the same shape and size (diameter of 8 mm and height of 2 mm), sterilized with 75% ethanol, and rinsed in phosphate-buffered saline (PBS) overnight. Four symmetrical small incisions were created on the back of each rat (female, Sprague Dawley, 180-210 g). The prepared samples were placed into the incisions, and then the skin was closed. After 7 days and 28 days, the rats were sacrificed, and the testing samples were excised with the adjacent tissues. The obtained material-tissue samples were embedded in paraffin, sectioned, and mounted onto slides. The acute inflammatory response and chronic inflammatory response were measured by both hematoxylin and eosin (H&E) staining, and toluidine blue (TB) staining. The stained slides were observed and analyzed by microscope.

**14. *In vitro*** **hemostasis performance**

The *in vitro* hemostasis properties of the cryogels were evaluated by red blood cell and platelet adhesion test, and dynamic whole blood clotting test [S6].

For blood cell and platelet adhesion tests, the cryogels were cut into disks with a height of 5 mm and a diameter of 8 mm, and then immersed into PBS for 1 h at 37 ℃. Following that, the ACD-whole blood was added dropwise onto the sample and then incubated for 5 min at 37 ℃. Platelet-rich plasma (PRP) was separated from the ACD-whole blood by centrifugation of blood at 116×g for 10 min. The PRP was then added dropwise onto the sample and incubated for 1 h at 37 ℃. All samples were then washed with PBS solution three times to remove the physical adhered blood cell and platelet and then fixed by 2.5% glutaraldehyde for another 2 h. After that, blood cells and platelets were dehydrated using 50%, 60%, 70%, 80%, 90%, and 100% ethanol solutions with time interval of 10 min. Finally, the samples were dried and observed using SEM.

For the whole blood clotting test, the cryogels were cut into cylindrical cryogel with a height of 3 mm and a diameter of 8 mm, and then the cylindrical cryogels were formed into shape-fixed situation. A volume of 50 μL of recalcified whole-blood solution (0.2 M CaCl_2_, 10 mM in the blood) was added onto the pre-warmed cryogels (37 ℃) in polypropylene tubes, respectively. Then, the tube was incubated at 37 ℃ for 30 s, 60 s, 120 s, and 180 s, respectively. The gauze and gelatin sponge were used as control groups. After the pre-set period, 10 mL of DI water was gently added to release unclotted blood without disturbing the clot. The absorbance of the supernatant was recorded at 540 nm by using a microplate reader (Molecular Devices). Three replicates were performed. The absorbance of 50 μL of recalcified whole-blood in 10 mL DI water was used as the reference value (positive control). The blood-clotting index (BCI) was calculated using equation:

BCI (%) = (I_s_ - I_o_) / (I_n_ - I_o_) × 100%,

where I_s_, I_n_, and I_o_ represent the absorbance of samples, positive control, and DI water, respectively.

**S1.15 *In vivo* hemostasis performance**

The in vivo hemostatic performance of GA/HD/MDP cryogel was evaluated by using the rat liver non-compressible hemorrhage model and coagulopathic rat liver non-compressible hemorrhage model [S10]. All animal studies were approved by the animal research committee of Xi’an Jiaotong University.

For rat liver non-compressible hemorrhage model, the rats (SD rats, weight 250-300 g, male) were randomly and equally divided into 4 groups. The animals were anesthetized and fixed on a surgical corkboard. The liver of the rat was exposed by abdominal incision, and serous fluid around the liver was carefully removed to prevent inaccuracies in the estimation of the blood weight obtained by the hemostatic samples. Then, created a 6 mm wide and 3 mm deep narrow wound. Immediately after wiping off the blood by using gauze, the shape-fixed dried cryogels or gelatin sponge was applied onto the site of the lesion. The group without hemostatic materials was used as blank control group. The hemostatic time and blood loss were recorded. Each group contains 6 rats.

For coagulopathic rat liver non-compressible hemorrhage model, the rats (SD rats, weight 250-300 g, male) with coagulopathy were prepared by injection of a clotting factor inhibitor. First, rivaroxaban solution (1 mg/mL) was prepared in a mixture of water and ethanol (10:9 v/v) with the help of ultrasound. Then, the rats were given 3 mg per 1 kg by gavage. After 90 min, the animals were anesthetized and fixed on a surgical corkboard. The liver of the rat was exposed by abdominal incision, and serous fluid around the liver was carefully removed to prevent inaccuracies in the estimation of the blood weight obtained by the hemostatic samples. Then, create a 6 mm wide and 3 mm deep narrow wound on rats. Immediately after wiping off the blood by using gauze, the shape-fixed dried cryogels or gelatin sponge was applied onto the site of the lesion. The group without hemostatic materials was used as blank control group. The hemostatic time and blood loss were recorded. Each group contains 6 rats.

**S1.16 *In vivo* wound healing performance**

All animal experiments were approved by the institutional review board of Xi’an Jiaotong University. Firstly, type 1 diabetes was induced in male Kunming mice using streptozotocin (100 mg/kg) according to previous literature [S11]. Three days after STZ administration, the blood glucose level of the mice was measured by the tail vein glucose meter (Yuwell, China), and the establishment of diabetes was determined when the blood glucose level of the mice exceeded 16.7 mmol/L. All mice were randomly and equally divided into 5 groups: Tegaderm^TM^ film, GA/HD/MDP0, GA/HD/MP2, GA/HD/MDP2, and GA/HD/MDP2+NIR groups. Each group contained 15 mice.

The mice were anesthetized, and then the dorsal region of mouse above the tail but below the back was shaved for further surgery. Two full thickness wounds with a diameter of 7 mm were made on each side of the mouse midline, and 10 μL MRSA (10^8^ CFU/mL) was injected into the wound to establish infection. Subsequently, different groups of dressings were applied to the wound site. For wound area monitoring, on the 5th, 10th, and 21st day, the mice were anesthetized, and then the wound area was photographed. The wound closure area was calculated by Image J. Wound closure ratio (%) was calculated by the following equation:

Wound closure ratio (%) = (A_0_ - A_n_) / A_0_ × 100%,

Where A_0_ and A_n_ represent the wound area of Day 0, and Day n (n = 5, 10, 21), respectively.

To evaluate the epidermal regeneration and inflammation in the wound area, the collected samples on the 5th, 10th, and 21st day were fixed with 4% paraformaldehyde for 1 hour, embedded in paraffin, and then cross-sectioned into 4 μm thickness slices. The obtained slices were then stained by Hematoxylin-Eosin (Beyotime, China) and Masson. All slices were analyzed and photographed by a microscope (IX53, Olympus, Japan). Meanwhile, CD80 and CD206 immunofluorescence staining was performed to observe the inflammatory response of the wounds in each group. The angiogenesis of wounds in each group was observed by VEGF immunofluorescence staining.

**S2 Results**

| 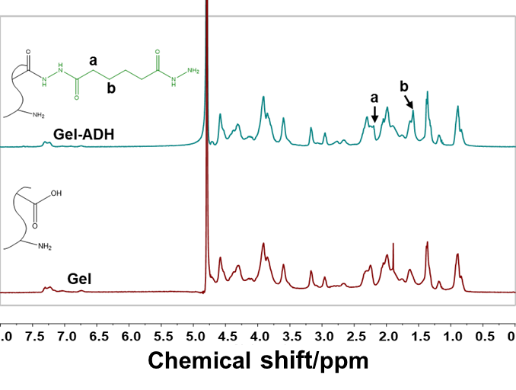  **Fig. S1** ^1^H NMR spectra of Gel-ADH (GA) **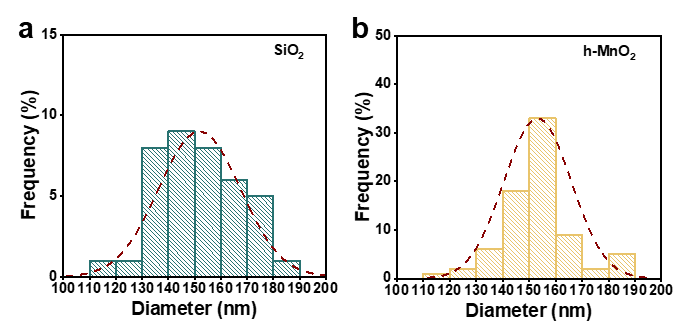**  **Fig. S2** The particle size distribution of (**a**) SiO_2_ and (**b**) h-MnO_2_ |
| --- |
|  |
|  |
| **** |
| **Fig. S3** The concentration of DFO before and after encapsulation |
| **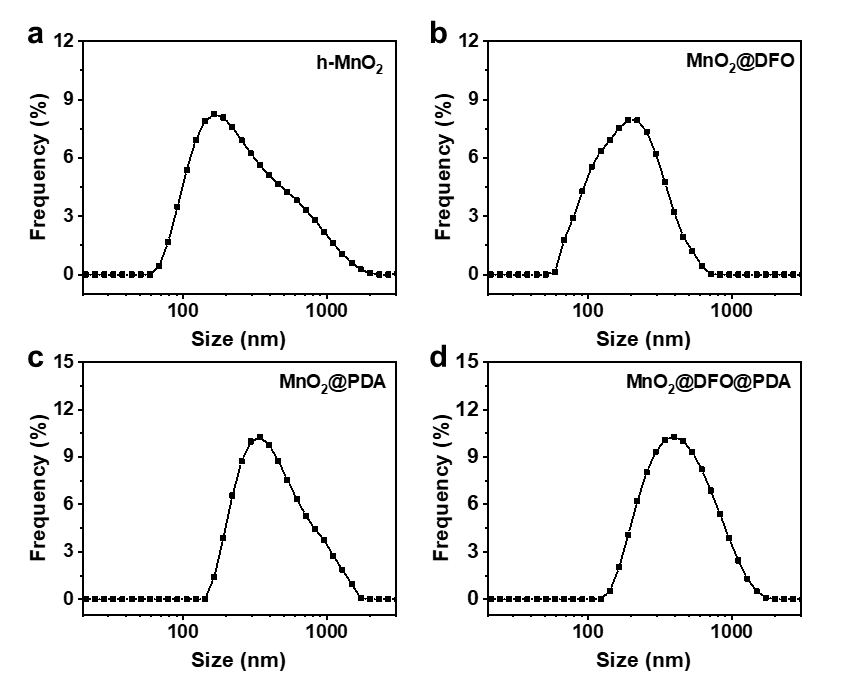** |
| **Fig. S4** The particle size distribution of (**a**) h-MnO_2_, (**b**) MnO_2_@DFO, (**c**) MnO_2_@PDA, and (**d**) MDP  **Table S1** The concentration of GA/HD cryogels components  **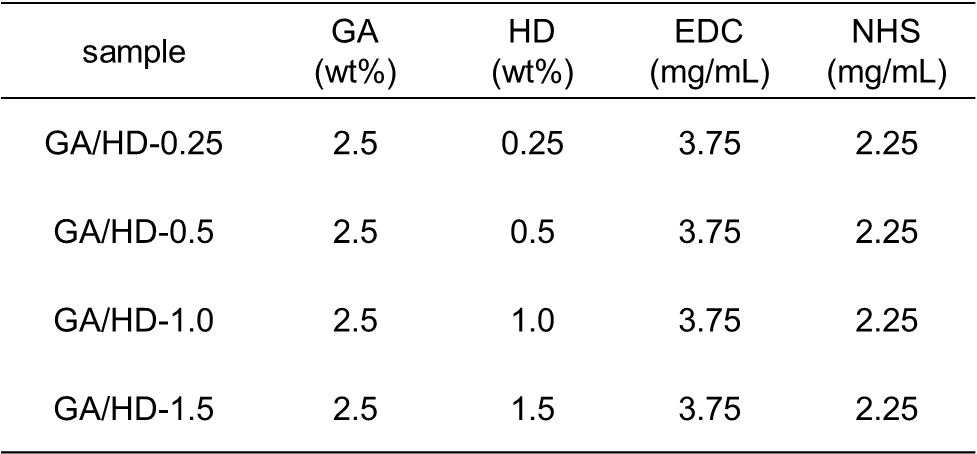** |
| When the HD concentration was 0.25 wt%, it was difficult to form a cryogel with stable mechanical properties due to the low crosslinking degree. Therefore, in subsequent experiments, only three concentrations of 0.5, 1.0, and 1.5 wt% were tested.  **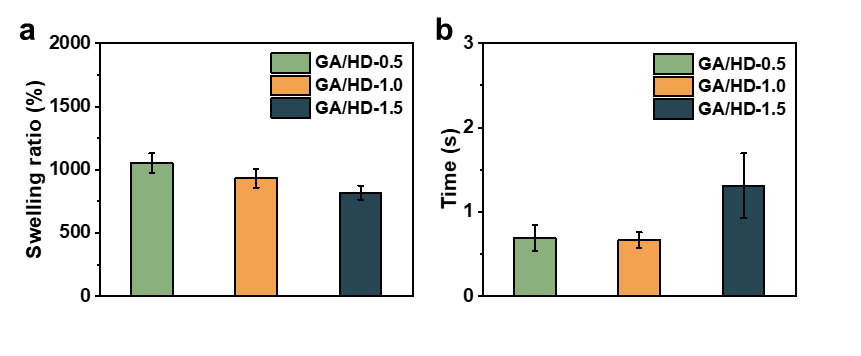**  **Fig. S5** (**a**) Swelling ratios of GA/HD cryogels. (**b**) Absorption time of GA/HD cryogels  **Supplementary References**   1. Y. Du, L. Li, H. Peng, H. Zheng, S. Cao et al., A spray-filming self-healing hydrogel fabricated from modified sodium alginate and gelatin as a bacterial barrier. Macromol. Biosci. **20**, 1900303 (2020). <https://doi.org/https://doi.org/10.1002/mabi.201900303> 2. L. Yang, Y. Sun, Q. Zou, T. Lu, W. Wang et al., Clean version: Electrospun fibrinogen scaffolds from discarded blood for wound healing. J. Biomed. Mater. Res. Part B Appl. Biomater. (2021). <https://doi.org/10.1002/jbm.b.34777> 3. G. Yang, L. Xu, Y. Chao, J. Xu, X. Sun et al., Hollow mno(2) as a tumor-microenvironment-responsive biodegradable nano-platform for combination therapy favoring antitumor immune responses. Nat. Commun. **8**, 902 (2017). <https://doi.org/10.1038/s41467-017-01050-0> 4. J. Li, F. Han, J. Ma, H. Wang, J. Pan et al., Targeting endogenous hydrogen peroxide at bone defects promotes bone repair. Adv. Funct. Mater. **32**, 2111208 (2022). <https://doi.org/https://doi.org/10.1002/adfm.202111208> 5. L. Kong, Z. Wu, H. Zhao, H. Cui, J. Shen et al., Bioactive injectable hydrogels containing desferrioxamine and bioglass for diabetic wound healing. ACS Appl Mater Interfaces **10**, 30103-30114 (2018). <https://doi.org/10.1021/acsami.8b09191> 6. Y. Huang, L. Bai, Y. T. Yang, Z. Yin, B. Guo, Biodegradable gelatin/silver nanoparticle composite cryogel with excellent antibacterial and antibiofilm activity and hemostasis for pseudomonas aeruginosa-infected burn wound healing. J. Colloid Interface Sci. **608**, 2278-2289 (2022). <https://doi.org/https://doi.org/10.1016/j.jcis.2021.10.131> 7. S. Zhu, M. Li, Z. Wang, Q. Feng, H. Gao et al., Bioactive glasses-based nanozymes composite macroporous cryogel with antioxidative, antibacterial, and pro-healing properties for diabetic infected wound repair. Adv. Healthcare Mater. **12**, 2302073 (2023). <https://doi.org/https://doi.org/10.1002/adhm.202302073> 8. J.H. He, Z.L. Li, J.X. Wang, T. Li, J. Chen et al., Photothermal antibacterial antioxidant conductive self-healing hydrogel with nitric oxide release accelerates diabetic wound healing. Composites, Part B **266**, 110985 (2023). <https://doi.org/https://doi.org/10.1016/j.compositesb.2023.110985> 9. Y.T. Yang, M. Li, G.Y. Pan, J. Chen, B. Guo, Multiple stimuli-responsive nanozyme-based cryogels with controlled NO release as self-adaptive wound dressing for infected wound healing. Adv. Funct. Mater. **33**, 2214089 (2023). <https://doi.org/https://doi.org/10.1002/adfm.202214089> 10. M. Li, Q. Dai, S. Zhu, Q. Feng, Z. Qin et al., An ultrafast water absorption composite cryogel containing iron-doped bioactive glass with rapid hemostatic ability for non-compressible and coagulopathic bleeding. Chem. Eng. J. **469**, 143758 (2023). <https://doi.org/https://doi.org/10.1016/j.cej.2023.143758> 11. J.Y. Chen, J.H. He, Y.T. Yang, L. Qiao, J. Hu et al., Antibacterial adhesive self-healing hydrogels to promote diabetic wound healing. Acta Biomater. **146**, 119-130 (2022). <https://doi.org/https://doi.org/10.1016/j.actbio.2022.04.041> |
